# Verrucones A–H, Non-Acetate Starter Aromatic Polyketides Discovered by Heterologous Expression of a Type II PKS Gene Cluster

**DOI:** 10.3390/microorganisms14081669

**Published:** 2026-07-30

**Authors:** Xingkun Hao, Ming Yang, Ping Yan, Qiyao Shen, Yang Liu, Youming Zhang, Xiaoying Bian, Haibo Zhou

**Affiliations:** Helmholtz International Lab for Anti-Infectives, Shandong University-Helmholtz Institute of Biotechnology, State Key Laboratory of Microbial Technology, Shandong University, Qingdao 266237, China

**Keywords:** type II PKS, aromatic polyketide, KAS III, genome mining, heterologous expression

## Abstract

Genome mining of the marine-derived *Streptomyces* sp. S42 uncovered a type II polyketide synthetase (T2 PKS) biosynthetic gene cluster (BGC) harboring a gene for 3-ketoacyl-ACP synthase III (KAS III), a hallmark of non-acetate starter unit incorporation, suggesting that the BGC may produce previously unidentified aromatic polyketides. Heterologous expression and promoter engineering of this prioritized BGC in host *Streptomyces albus* J1074 activated the biosynthetic pathway, leading to the isolation of eight new polycyclic aromatic derivatives, verrucones A–H (**1**–**8**). Comprehensive structural elucidation via NMR and HRESIMS revealed that these compounds feature either a 2-methylbutyryl or an isobutyryl starter unit and can be classified into three distinct skeletal types. Based on these findings and bioinformatic analysis, a plausible biosynthetic pathway for **1**–**8** involving divergent spontaneous cyclization from a common nascent polyketide intermediate was proposed. Among the isolated compounds, **1**–**5** exhibited inhibitory activity against several protein tyrosine phosphatases (PTPs) with IC_50_ values ranging from 1.84 μM to 24.82 μM. This study presents a successful case study demonstrating that combining KAS III-targeted genome mining with heterologous expression is a viable approach for discovering non-acetate-primed aromatic polyketides.

## 1. Introduction

Aromatic polyketides from actinobacteria represent a class of natural products with potential for clinical application due to their potent biological activities. Representative aromatic polyketide drugs include doxorubicin and tetracycline, which are approved anticancer and antibacterial drugs, respectively [1,2]. In addition, aromatic polyketides exhibit many other biological activities, such as antiviral, antioxidant, and enzyme inhibition activities, making them invaluable leads in drug discovery and development [3]. Among these diverse bioactivities, the inhibitory effects against protein tyrosine phosphatases (PTPs) have garnered increasing attention [4,5]. Protein tyrosine phosphatases jointly regulate tyrosine phosphorylation in cellular processes. Modulating PTP activity holds promise for the treatment of various human diseases, such as type II diabetes, obesity, cancer, and inflammatory diseases [6,7,8,9]. Therefore, discovery of new aromatic polyketides for PTP inhibitor screening is of significant value for the treatment of related diseases.

Bacterial aromatic polyketides are generally biosynthesized by type II polyketide synthase (T2 PKS) systems [3,10]. A defining feature of these systems is the so-called “minimal PKS”, which catalyzes iterative decarboxylative condensations between an acyl-CoA starter unit and malonyl-CoA extender units, thereby generating a growing polyketide chain. Subsequently, ketoreductases (KRs) together with aromatases (AROs) and cyclases (CYCs) coordinate the regioselective folding and cyclization of the linear intermediate. In this process, the C-9 KR, along with specific ARO and CYC, is essential for proper aromatization; otherwise, the highly reactive nascent polyketide chain will undergo intramolecular spontaneous aberrant cyclization, forming shunt products [11]. Finally, the normal cyclized product then serves as a common precursor, which undergoes a series of tailoring reactions such as redox reactions, group transfers, and rearrangements to yield the mature polycyclic aromatic products [12].

Based on the biosynthetic logic outlined above, the structural diversity of aromatic polyketides is largely governed by variations in starter units, cyclization patterns, and tailoring modifications. While the vast majority of aromatic polyketides are initiated with acetyl-CoA, a growing number of biosynthetic pathways have been found to employ nonacetate primers, including benzoate, propionate, butyrate, and even amino acids derived units [13,14,15]. Unlike canonical acetyl-CoA primed formation, the biosynthesis and incorporation of such atypical starters often involve the additional 3-ketoacyl-ACP synthase (KAS III) that selectively recognizes and loads the alternative starter units into the minimal PKS for elongation. Consequently, KAS III-encoding genes within a biosynthetic gene cluster (BGC) can serve as a valuable genomic marker for the targeted discovery of non-acetate primed aromatic polyketides.

With the expanding availability of genomic data, increasing evidence indicates that actinobacteria genomes harbor an abundance of T2 PKS BGCs, many of which remain silent under standard laboratory cultivation conditions [16]. To unlock this hidden reservoir, genome mining combined with heterologous expression has emerged as one of the most effective and widely adopted strategies [17,18]. Bioinformatics platform antiSMASH [19] assists in the prediction of T2 PKS BGCs from genomic sequences, serving as the basis for subsequent prioritization and dereplication, which facilitated the targeted selection of promising gene clusters. Subsequent heterologous expression of entire BGCs in well-characterized heterologous hosts effectively bypasses the regulatory constraints of the native producer and often leads to the activation of silent biosynthetic pathways [20,21]. This approach has proven remarkably successful by our research along with others, leading to the discovery of numerous novel aromatic polyketides from previously uncharacterized clusters and substantially expanding the chemical space of bacterial type II PKS products [21,22,23,24,25].

During our ongoing genome mining efforts to discover new bioactive aromatic polyketides from actinobacteria [22,26], the marine-derived *Streptomyces* sp. S42 attracted our attention. Bioinformatic analysis revealed that its genome contains a T2 PKS BGC, designated *ver*, which harbors a KAS III encoding gene, suggesting a non-acetate starter unit incorporation in its corresponding products. Compared with homologous gene clusters [27,28], the *ver* cluster stands out by possessing several oxidoreductases with lower similarity alongside the conserved core genes, suggesting a potential for producing new metabolites. Heterologous expression of this BGC in the host *Streptomyces albus* J1074 successfully activated the biosynthetic pathway, leading to the identification of eight new aromatic polyketides, verrucones A–H (**1**–**8**). These compounds not only feature unique 2-methylbutanyl or isobutyryl moieties as their starter units but also display diverse cyclization patterns, giving rise to three distinct skeletal frameworks. Details of the structure elucidation, biological evaluation, and plausible biosynthetic pathways of these new compounds are reported herein.

## 2. Materials and Methods

### 2.1. General Experimental Procedures

UV spectra and UHPLC-HRESIMS data were acquired using a Thermo Scientific™ Dionex™ Ultimate 3000 system (Thermo Fisher Scientific, Waltham, MA, USA) coupled with a Bruker microTOF Q III mass spectrometer (Impact HD) (Bruker Corporation, Rheinstetten, Germany) equipped with a standard ESI ion source in positive electrospray ionization mode. A Thermo Scientific™ Acclaim™ C18 column (2.1 × 100 mm, 2.2 μm) (Thermo Fisher Scientific, Waltham, MA, USA) was used. The elution program was set as follows: solvent A, H_2_O with 0.1% formic acid (FA); solvent B, 0.1% FA in acetonitrile (ACN), 0–3 min 5% B, 3–18 min, 5–95% B, 18–22 min, 95% B, and 23–25 min, 5% B, with flow rate of 0.3 mL/min. Column chromatography (CC) was performed using silica gel (200–300 mesh, Qingdao Marine Chemical Factory, Qingdao, China). Semipreparative HPLC was conducted on either a Shimadzu Essentia LC-20AT (SHIMADZU Corporation, Kyoto, Japan) or an Agilent 1260 Infinity II LC system (Agilent Technologies Inc., Santa Clara, CA, USA). Separations utilized reverse-phase C18 columns: Agilent ZORBAX SB-C18 (9.4 × 250 mm, 5 μm) (Agilent Technologies Inc., Santa Clara, CA, USA) or YMC-pack ODS-A (10 × 250 mm, 5 μm) (YMC CO., Ltd., Kyoto, Japan), with a flow rate of 2 mL/min. NMR spectra (^1^H, ^13^C, and 2D NMR data) were recorded on a Bruker Avance NEO 600 MHz spectrometer (Bruker Corporation, Rheinstetten, Germany). Chemical shifts (*δ*) are reported in ppm relative to the residual solvent signals: DMSO-*d*_6_ (*δ*_H/C_ 2.50/39.52). Structural assignments were supported by analysis of 2D NMR data (gCOSY, gHSQC, and gHMBC).

### 2.2. Strain Isolation, Identification, and Genome Sequencing

The S42 strain was isolated from a sea mud sample collected from the coastal area of Aoshanwan, Qingdao, Shandong Province, China (36°20′ N, 120°43′ E) in September 2018. 16S rRNA gene sequence analysis identified its closest phylogenetic relative as *Streptomyces verrucosisporus*, sharing 98.71% sequence identity. The S42 strain grown on salinated ISP2 plates was inoculated into 50 mL of salinated YEME medium (Table S1) in 250 mL flasks. The culture was incubated at 30 °C at 200 rpm for 36 h for genomic DNA extraction according to a previously described method [29]. Whole-genome sequencing was conducted by Novogene using the PacBio platform. The biosynthetic gene clusters within the S42 genome were analyzed by antiSMASH (https://antismash.secondarymetabolites.org) (accessed on 20 November 2018) [19]. Protein similarity searches were performed using the Basic Local Alignment Search Tool (https://blast.ncbi.nlm.nih.gov/Blast.cgi) (accessed on 25 November 2018) [30] against the NCBI database.

### 2.3. Direct Cloning and Heterologous Expression of Ver Gene Cluster

The pBeloBAC11 vector was used to directly clone the 60 kb *ver* gene cluster by means of the ExoCET method [31]. The prepared genomic DNA (50 μg) above was digested with *Nde*I-*Hind*III, extracted with phenol/chloroform/isoamyl alcohol (25:24:1, pH 8.0), and precipitated with ethanol. The DNA was dissolved in ddH_2_O. The pBeloBAC11 vector carrying homology arms of *ver* genes was amplified with PCR using the 2× ApexHF FS PCR Master Mix (Accurate Biology, Qingdao, China). The digested genomic DNA (10 μg) and amplified pBeloBAC11 (1 μg) were assembled in vitro and then electroporated for homologous recombination. The recombinant plasmid pBAC-*ver* was identified with *Asc*I and *Kpn*I restriction analysis (Figure S2b). Thereafter, it was modified via linear plus circular homologous recombination (LCHR) to insert the *oriT-attP-phiC31* cassette from pR6K-oriT-phiC31 amplified by PCR (Table S2), forming pBAC-*ver*-apra-phiC31 for targeting in a heterologous host’s genome (Figure S2a). The correct plasmid pBAC-*ver*-apra-phiC31 was introduced into *S. albus* J1074, termed J1074-*ver*, via conjugation from *E. coli* ET12567/pUZ8002 according to the literature procedure [32].

### 2.4. Insertion of the Strong Constitutive Promoter kasOp*

The ermE*p-hyg-kasOp* cassette harboring 40 bp homology arms was amplified from the p15A-ermE*p-hyg-kasOp* plasmid using the 2× ApexHF FS PCR Master Mix (AG). The amplified cassette was subsequently inserted upstream of the *verA3* gene by LCHR, resulting in the construction of pBAC-ver-kasOp* (Figure S3).

### 2.5. Inactivation of “Core PKS” Genes

The core PKS genes *verA1*–*A3* were deleted utilizing the CcdB counterselection strategy [33], thereby avoiding the introduction of a new resistance gene. The DNA fragment *amp-ccdB* was amplified by PCR using the plasmid p15A-*amp-ccdB* as a template with primers that contained 40 bp homology arms and the *Pac*I restriction site (TTAATTAA). The amplified fragment was inserted into pBAC-*ver* by LCHR to replace *verA1*–*A3*, and positive clones were selected on ampicillin-containing LB plates (100 μg·mL^−1^) and verified by restriction digestion. The resulting plasmid was then digested with *Pac*I to remove the *amp-ccdB* cassette, and the product was ligated in vitro with T4 DNA ligase before being electroporated into *E. coli* GB2005. Recombinants were selected on antibiotic-free LB plates, and the resulting plasmid was extracted and confirmed by restriction digestion (Figure S3C), yielding the correct gene knockout plasmid designated pBAC-*ver*-ΔPKS.

### 2.6. Fermentation, Extraction, and Isolation of Compounds ***1***–***8***

For product detection, the mutant strains were grown on MS agar plates (containing 2% mannitol, 2% soya flour, and 2% agar). Three separate fermentations started from different single colonies were performed using YM medium in 250 mL flasks. After incubation at 30 °C and 200 rpm for 5 days, 2% (*v*/*v*) XAD-16 resin was added, and the mixture was further incubated for 3 days. Metabolites were then extracted from the resin with methanol and analyzed by LC-MS. For analytical measurements, three independently prepared sample sets derived from separate biological replicates were analyzed.

For large-scale fermentation, the J1074-ver-kasOp* strain was grown on MS agar plates. The colonies from this plate were inoculated into 50 mL of YM medium in 250 mL flasks. The culture was incubated at 30 °C and 200 rpm for 36 h to serve as the seed culture. Subsequently, 2% (*v*/*v*) of the seed culture was transferred into 50 mL of YM fermentation medium in 250 mL flasks. Following incubation at 30 °C and 200 rpm for 5 days, 2% (*v*/*v*) XAD-16 resin was added, and the mixture was incubated for another 3 days. Metabolites were then extracted from the XAD-16 resin using methanol and analyzed by LC-MS. The crude extracts obtained from 13.5 L fermentation were fractionated using normal-phase silica gel column chromatography and eluted with a stepwise gradient of dichloromethane (CH_2_Cl_2_) and methanol (MeOH) (100:0, 50:1, 40:1, 30:1, 20:1, 15:1, 10:1, 5:1, 2:1, *v*/*v*), yielding 9 fractions (Fr.1–Fr.9). Fr.4 was purified by semipreparative HPLC (ODS; 5 μm, 250 × 9.4 mm; ACN–H_2_O, 42:58, *v*/*v*; 2 mL/min) to afford compound **4** (8.7 mg). Fr.5 was purified under the same column conditions with ACN–H_2_O (33:67, *v*/*v*) to yield compound **2** (10.0 mg). Fr.6 was eluted by semipreparative HPLC with ACN–H_2_O (34:66, *v*/*v*) to yield compound **5** (2.1 mg). Fr.7 was separated into seven subfractions (Fr.7.1–Fr.7.7) by MPLC with a step gradient elution with ACN–H_2_O (15–100% in 40 min). Fr.7.4 was further purified by using semipreparative HPLC with 28% ACN–H_2_O to obtain **1** (5.2 mg) and **3** (3.5 mg). Fr.7.5 was purified on semipreparative HPLC (ODS; 5 μm, 250 × 10 mm) with 35% ACN–H_2_O to give **6** (5.0 mg), **7** (3.0 mg), and **8** (5.0 mg).

### 2.7. Bioactivity Assays

Cell Counting Kit (CCK-8) was used to evaluate the antitumor activity [34]. Compounds were dissolved in the cell-grade DMSO after accurately weighing to generate the 10 mM drug solution. The drug solution was further diluted to a tenfold detection concentration in cell culture medium. Six cell lines, including human breast cancer cell line (MCF-7), human liver cancer cell line (HepG2), human colorectal adenocarcinoma cell line (HT29), human promyelocytic leukemia cell line (HL60), Human acute T-cell leukemia cell line (Jurkat), and Human embryonic kidney cell line (293T) were used.

Four protein tyrosine phosphatases (PTPs), PTP1B, SHP1, SHP2, and TCPTP, were selected for inhibitory activity screening according to the previous report with slight adjustments [35]. The PTPs were first pre-incubated with the test compounds at 30 °C for 15 min. Subsequently, a reaction mixture (50 mM HEPES, 100 mM NaCl, 1 mM EDTA, 1 mM dithiothreitol) containing the enzyme–compound complex was incubated in 96-well plates at 30 °C for 15 min. The enzymatic reaction was initiated by adding 2 mM p-nitrophenyl phosphate (pNPP), followed by a further 15–30 min incubation at 37 °C, and terminated with 50 μL of 3 M NaOH. Sodium orthovanadate (Na_3_VO_4_) and DMSO were used as positive and negative controls, respectively. Since pNPP is dephosphorylated by PTPs to yield p-nitrophenol, the inhibitory activity could be assessed by measuring the absorbance at 405 nm, which reflects the amount of product formed. Compounds exhibiting an inhibition rate greater than 50% at 50 μg/mL were selected for further IC_50_ determination. Three independent biological replicates were conducted. And within each biological replicate, all samples were run in four technical wells to account for intra-assay variability. The percentage inhibition was calculated as [1 − (sample − blank)/(control − blank)] × 100%. The IC_50_ values were determined by fitting the dose–response curves using non-linear regression analysis with GraphPad Prism 6.0 and are expressed as mean ± standard deviation (SD) derived from three biological replicates.

## 3. Results

### 3.1. Identification of KAS III Containing Type II PKS BGC Ver

**Figure 1:**
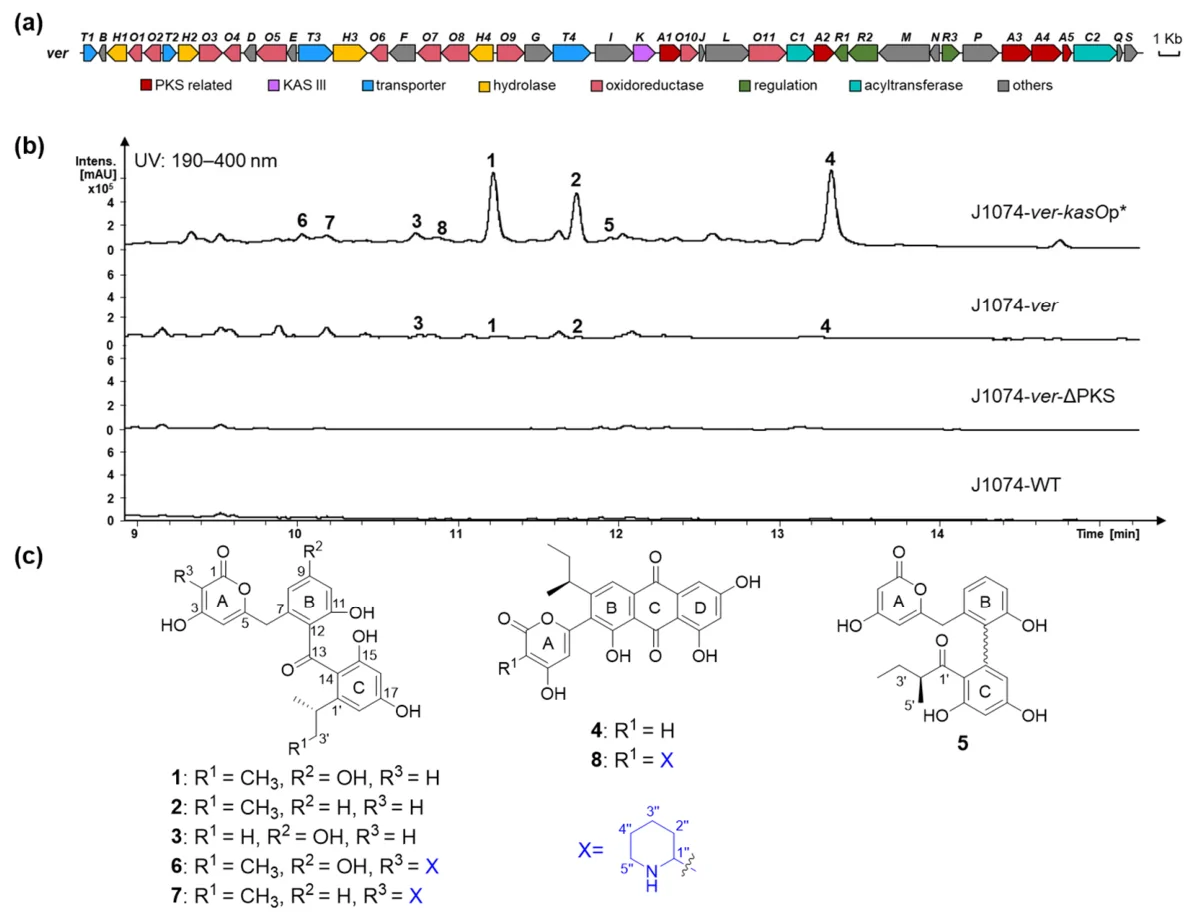
(**a**) Gene organization of the *ver* gene cluster. (**b**) Comparative metabolite analysis of the J1074-*ver* strain and the J1074-ver-kasOp* cultivated in YM medium. (**c**) Structures of **1**–**8**.

The presence of KAS III in type II polyketide gene clusters broadens the structural diversity of the resulting polyketides by enabling the incorporation of non-acetate starter units, which leads to variations in the aromatic initiation moieties [36]. Genomic analysis of our stored strains identified a type II PKS BGC *ver* (GenBase accession number: C_AA474359.1) harboring KAS III in *Streptomyces* sp. S42. AntiSMASH analysis indicates that only two known KAS III-containing type II PKS clusters *txn* [27] and *lld* [28] show 55% and 54% similarity to *ver*, respectively. Based on comparison with these characterized BGCs, the *ver* gene cluster was proposed to span approximately 48.9 kb, containing 43 open reading frames (Figure 1a). The PKS backbone-related genes of the *ver* cluster comprise “minimal PKS” *VerA3*–*A5* and aromatase/cyclase *VerA1* but lack a ketoreductase. Additionally, the *ver* gene cluster also contains several other genes, including putative oxidoreductases and regulatory factors. (Figure S1, Table S4), which show low sequence homology to those in the other two BGCs, suggesting its potential to generate novel non-acetate primed products.

### 3.2. Identification of Verrucones by Heterologous Expression

To activate the *ver* gene cluster and obtain its biosynthetic products, a genomic fragment encompassing the entire cluster was cloned into the pBeloBAC11 vector in *E. coli* using the ExoCET method [31] (Figure S2) and then heterologously expressed in *S. albus* J1074. Fermentation of the engineered strain J1074-*ver* and HPLC analysis of the crude extract revealed several new peaks, indicating the activation of target products, though production was initially low (Figure 1b). To further improve the yield of the products, a constitutive strong promoter kasOp* was inserted upstream of the core gene *verA3* (Figure S3). HPLC analysis indicated that the yield of the target compounds was significantly increased (Figure 1b). Deletion of the “minimal PKS” genes *verA3*–*A5* completely abolished all observed peaks, confirming that the *ver* gene cluster is responsible for the biosynthesis of these compounds.

To obtain enough amounts of the corresponding products for structural characterization, a scale-up fermentation (13.5 L) of the engineered strain J1074-ver-kasOp* was performed. The crude extract was then fractionated by silica gel column chromatography and further purified by semipreparative HPLC, which led to the isolation of eight compounds, named verrucones A–H (**1**–**8**) (Figure 1c).

**Figure 2:**
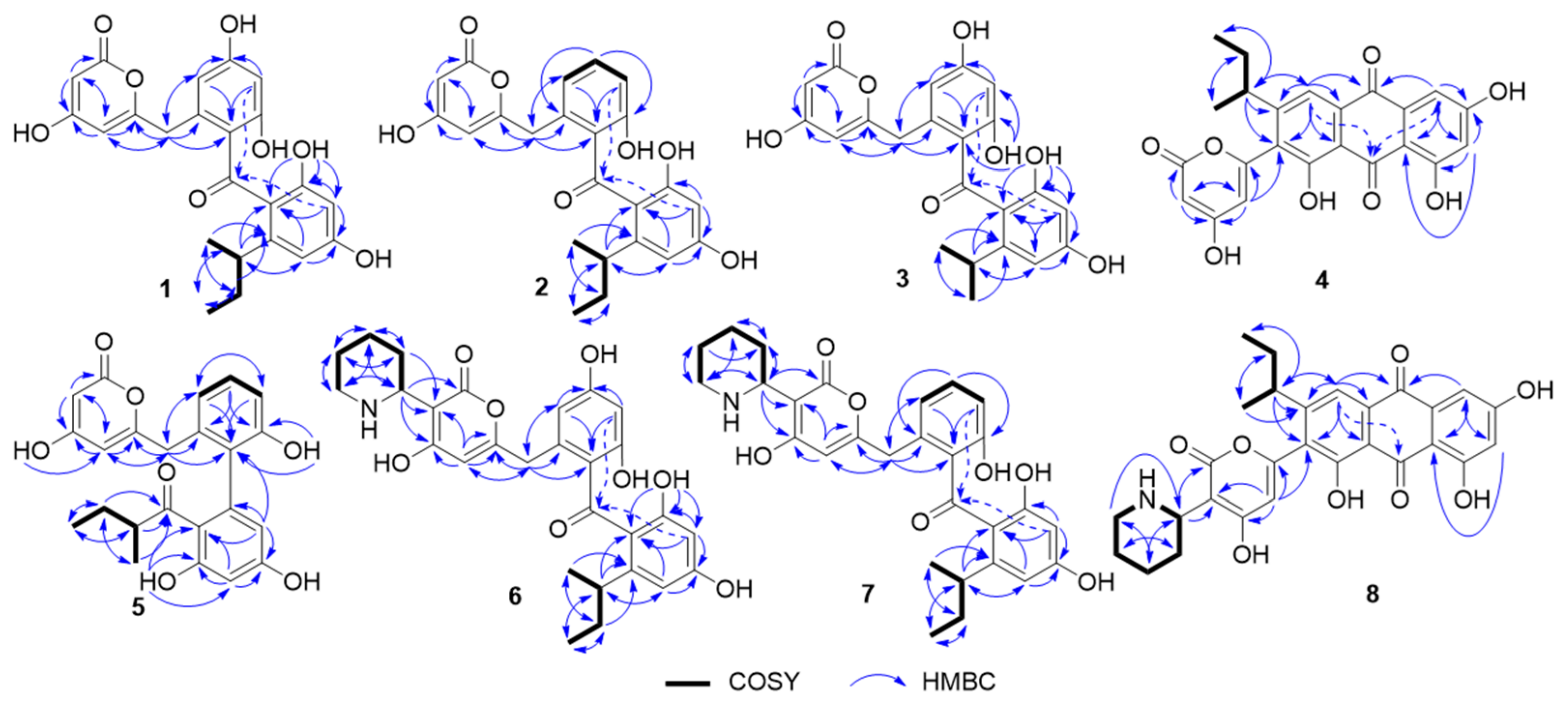
Key COSY and HMBC correlations of **1**–**8**.

Verrucones A–C (**1**–**3**) were isolated as brown solids, with the molecular formulas C_23_H_22_O_8_, C_23_H_22_O_7_, and C_22_H_20_O_8_, respectively, as deduced from their HRESIMS ions at *m*/*z* 427.1387, 411.1438, and 413.1231 [M + H]^+^. The ^1^H, ^13^C, DEPT, and HSQC NMR data of **1** (Table 1, Figures S6–S9) revealed the presence of one ketone carbonyl (*δ*_C_ 199.8), eleven non-protonated carbons with seven oxygenated aromatic carbons (*δ*_C_ 170.3, 164.9, 163.7, 161.0 (2C), 159.9, 157.0), six sp^2^ methines (*δ*_H/C_ 6.21/101.7, 6.15/104.3, 6.14/110.0, 6.09/100.2, 5.66/100.8, 5.19/88.4), one sp^3^ methine (*δ*_H/C_ 2.48/36.6), two methylenes (*δ*_H/C_ 3.57/36.8; 1.43,1.33/30.4), and two methyl groups (*δ*_H/C_ 0.97/21.4, 0.66/12.2). Four structural fragments, including a pyrone moiety (ring A), two tetrasubstituted benzene rings (rings B and C), and a butyl side chain, were readily established by analysis of HMBC and COSY correlations (Figure 2, Figures S10 and S11). Key HMBC correlations from H-6 to C-4, C-5, C-7, C-8, and C-12 confirmed the connection of rings A and B through C-6 (Figure 2 and Figure S11). The weak long-range correlations from H-10 and H-16 to C-13 supported the location of C-13 between rings B and C. Similarly, the attachment of the *sec*-butyl side chain at C-1′ was determined by the key HMBC correlations from H-2′ and H-5′ to C-1′. The ^1^H and ^13^C NMR data of **2** and **3** were highly similar to those of **1** (Table 1, Figures S12, S13, S18 and S19), with **2** lacking one hydroxy group and **3** missing one methylene moiety. Further analysis of successive COSY correlations from H-8 (*δ*_H_ 6.71)/H-9 (*δ*_H_ 7.19)/H10 (*δ*_H_ 6.74) in **2** (Figure 2 and Figure S16) and H-3′ (*δ*_H_ 1.01)/H-2′ (*δ*_H_ 2.76)/H-4′ (*δ*_H_ 1.01) in **3** (Figure 2 and Figure S21) indicates that the tetrasubstituted ring B in **1** becomes a trisubstituted benzene ring in **2**, and the *sec*-butyl side chain is replaced by isopropyl in **3**, respectively. The detailed planar structures of **2**–**3** were further confirmed by the COSY and HMBC correlations (Figure 2). According to bioinformatic analysis and the established biosynthesis of TXN**4a** from L-isoleucine (confirmed by ^13^C-labeled precursor feeding experiments [27]), we propose that the *sec*-butyl is derived from L-isoleucine, indicating that the absolute configuration at C-2′ is *S*. A literature search using SciFinder revealed that compounds **1**–**3** differ from the known compound SEK15 in that they bear unique 2-methylbutyryl or iso-butyryl side chains [37,38].

Verrucone D (**4**) was isolated as a yellow solid. Its molecular formula was determined to be C_23_H_18_O_8_ based on the HRESIMS ion at *m*/*z* 423.1060 [M + H]^+^. The one-dimensional (1D) NMR data of **4** (Table 2, Figures S23 and S24) showed that it shares the same pyrone ring (ring A) and *sec*-butyl group as **1**. The remaining ^1^H and ^13^C NMR signals exhibited characteristic resonances consistent with an anthraquinone skeleton, including two carbonyl carbons at *δ*_C_ 189.0 and 181.1, twelve aromatic carbons in the range of *δ*_C_ 108.1–166.4, two meta-coupled aromatic protons at *δ*_H_ 7.13 (d, *J* = 2.0 Hz) and 6.58 (d, *J* = 2.0 Hz), and one singlet aromatic proton at *δ*_H_ 7.41. The linkage of the above three units, as well as the complete structure of **4**, was further established by 2D NMR correlations (Figure 2 and Figures S26–S28).

Verrucone E (**5**) was isolated as a yellow solid, sharing the same molecular formula C_23_H_22_O_7_ with **2**, as evidenced by the HRESIMS ion at *m*/*z* 411.1428 [M + H]^+^. Although compounds **5a** and **5b** exhibited different HPLC retention times, they interconvert rapidly during the separation process. Therefore, the NMR spectrum of the mixture was acquired to elucidate their structures. The 1D NMR data of **5a** and **5b** (Table 2, Figures S29 and S30) were similar to those of **2**, especially the chemical shifts in C-1–C-12, suggesting that rings A and B remain intact. Key HMBC cross-peak from H-14 to C-12 indicates the direct connection between rings B and C, while correlations from 17-OH and H-2′/H-3′/H-5′ to C-1′ confirmed the attachment of the 2-methylbutyryl group at C-18 (Figure 2 and Figure S33). Thus, compound **5** was determined to have a biphenyl skeleton; however, the absolute configuration of its axial chirality has not yet been established because of the rapid interconversion of **5a** and **5b**.

Verrucone F–H (**6**–**8**) were obtained as brown solids, and their molecular formulas were assigned to be C_28_H_32_NO_8_, C_28_H_32_NO_7_, C_28_H_28_NO_8_ from HRESIMS peaks at 510.2109, 494.2164, 506.1797 [M + H]^+^, respectively. The 1D NMR data of **6** were similar to those of **1** (Table 1 and Table 3, Figures S34 and S35), except for five additional carbon signals in the range of *δ*_C_ 22.1–53.7 (C-1″–C-5″), indicating the presence of a piperidine moiety. This assignment was further supported by successive COSY correlations from H-1″ to H-5″ (Figure 2 and Figure S38). HMBC correlations from H-1″ to C-1 and C-2 established that the piperidine ring is located at C-2 (Figure 2 and Figure S39). Similarly, compounds **7**–**8** were determined to be piperidine-substituted derivatives of **2**–**3**, respectively, as deduced from their molecular formulas and further confirmed by 2D NMR (Figure 2, Figures S43–S45 and S49–S51).

### 3.3. Proposed Biosynthetic Pathway of Verrucones

**Figure 3:**
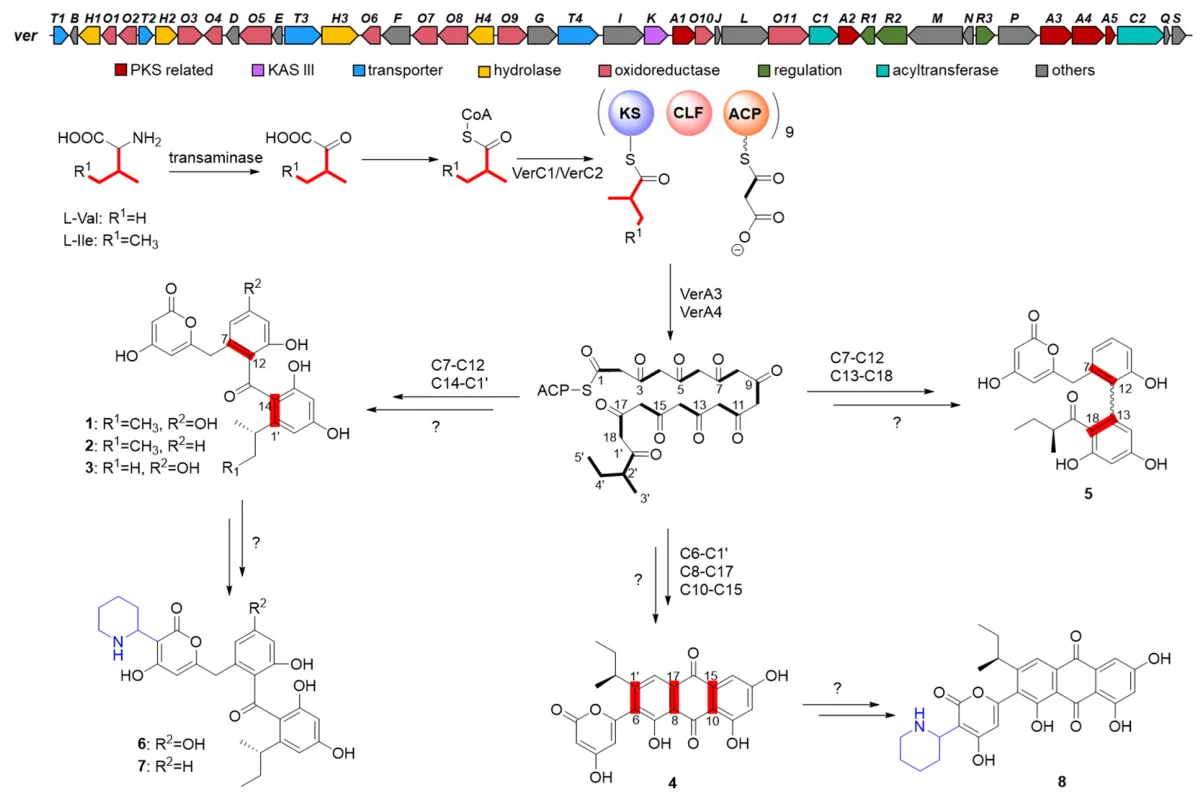
Proposed biosynthetic pathway of verrucones A–H (**1**–**8**).

Based on bioinformatic analysis of the *ver* gene cluster and the structural characterization of verrucones A–H (**1**–**8**), a plausible biosynthetic pathway for these compounds was proposed (Figure 3). Following deamination and decarboxylation, L-isoleucine or valine is proposed to be converted by a CoA transferase into its CoA ester, generating the corresponding acyl-CoA precursor [27]. The ATs encoded by *verC1* and *verC2* then are thought to specifically recognize the 2-methylbutyryl-CoA and isobutyryl-CoA as starter units and transfer them to the ACP encoded by *verA5*. Subsequently, the KS_α_ and KS_β_ encoded by *verA3* and *verA4* could catalyze the condensation of the initiation unit and nine molecules of malonyl-CoA, thereby forming the nascent polyketide chain. This reactive intermediate may then undergo divergent spontaneous cyclization pathways, ultimately giving rise to three distinct skeletal frameworks, as observed in verrucones A–H (**1**–**8**). Among them, the cyclization patterns of C7-C12 and C14-C1′ form **1**–**3**, a combination of C6-C1′, C8-C17, and C10-C15 cyclization generates **4**, whereas C7-C12 and C13-C18 cyclization yield **5**. Subsequently, the hydroxyl group at C-9 of **1** is removed either enzymatically by a dehydroxylase or spontaneously, resulting in **2**. Additionally, compounds **1**, **2**, and **4** were converted to their piperidine derivatives **6**–**8**, respectively. The detailed mechanism of this transformation, as well as the complete biosynthetic pathway, remains to be established by further studies.

### 3.4. Bioactivities of Verrucones

In the bioassay, only compound **3** exhibited weak cytotoxicity against the human breast cancer cell line MCF-7, with an IC_50_ of 16.39 μM, but showed no cytotoxicity against the normal human kidney cell line 293T (Table S5). Compounds **1**–**5** displayed inhibitory activities against PTP1B and TCPTP, with IC_50_ values ranges of 1.84–12.38 μM and 1.84–10.24 μM, respectively. Compounds **1**, **2**, and **4** also inhibited SHP1, whereas compounds **1** and **4** were active against SHP2 (Table 4). Compounds **6**–**8** were inactive against all tested PTPs.

## 4. Discussion and Conclusions

KAS III enzymes are normally involved in the initiation of type II PKSs assembly and exhibit broad substrate flexibility, allowing them to utilize diverse non-acetate starter units [13,36]. Through genome mining of the marine-derived *Streptomyces* sp. S42, we identified a T2 PKS BGC (*ver*) carrying KAS III-encoding genes. The presence of the KASIII enzyme in the *ver* cluster suggests that its products may have non-acetate start units. Analysis of *ver* revealed that it contains two genes *verC1* and *verC2* encoding two separate ATs. AntiSMASH analysis predicts that these two ATs recognize 2-methylbutyryl-CoA and isobutyryl-CoA, respectively. These prediction results are consistent with the two non-acetate starting units in the structure of the isolated compounds, verrucones A–H (**1**–**8**). We hypothesize that the 2-methylbutyryl and isobutyryl groups originate from the deamination and decarboxylation of L-isoleucine and L-valine, respectively. The involvement of KASIII in the initiation of type II PKSs increases the structural complexity and diversity of aromatic polyketide products.

The correct cyclization of T2 PKS products is dependent on the action of a C-9 KR in conjunction with specific AROs and CYCs [11]. In the absence of these auxiliary proteins, the highly reactive nascent polyketide chain fails to undergo regioselective aromatization and instead proceeds via spontaneous, non-enzymatic cyclization [38]. Consistent with this paradigm, the *ver* BGC does not encode a C-9 KR, and its products exhibit three distinct skeletal types, possibly arising from spontaneous cyclization. From the perspective of type II PKS evolution, the absence of KR effectively reverts the system to a more primitive evolutionary state, where the minimal PKS merely drives carbon chain elongation, while all subsequent cyclization cascades are spontaneous [39]. This also demonstrates the flexibility of PKS as a source of structurally varied natural products.

In comparison with the homologous gene clusters *txn* [27] and *lld* [28], the *ver* cluster exhibits conserved minimal PKS core genes. However, it differs by harboring several oxidoreductase genes (*verO4*, *verO8*, *verO10*, and *verO11*) while being devoid of glycosyltransferase genes. This compositional feature implies that the *ver* biosynthetic pathway may produce non-glycosylated products, and the presence of oxidoreductases further hints at potential oxidative modifications. Consequently, *ver* potentially generating products with unique starter units and diverse cyclization patterns that are structurally different from previously characterized natural products. As a result, the compounds we isolated display variability in post-PKS modifications, notably at C-9 hydroxylation and the introduction of a piperidine unit. The piperidine moiety in natural products generally originates from the incorporation of L-pipecolic acid, which is an intermediate in L-lysine metabolism [40]. Since the *ver* cluster does not contain a homologous gene encoding lysine cyclodeaminase, we speculate that the gene responsible for catalyzing the formation of piperidine may be present in the host genome.

Verrucones A–E (**1**–**5**) exhibited differential inhibitory potencies against PTP targets. Specifically, a comparison of compounds **1** and **2** revealed that the absence of the hydroxyl group at C-9 position attenuates the inhibitory activity. Similarly, comparison of **1** and **3** indicated that the 2-methylbutyryl group influences activity, with compound **3** being less potent than **1**. Notably, compound **4** was the most potent among the tested compounds, suggesting that the anthraquinone structure may contribute favorably to the PTP inhibitory activity, consistent with a previous report [5]. In contrast, compounds **6**–**8** were inactive, implying that C-2 piperidine substitution significantly diminishes PTPs’ inhibitory potency. Although compound **4** displayed promising activity, its selectivity remained insufficient. Future structural modifications potentially via synthetic biology approaches could be employed to improve its selectivity profile. Collectively, these findings provide a basis for the rational design of selective PTP inhibitors.

In summary, genome mining of *Streptomyces* sp. S42 led to the discovery of a type II PKS BGC, designated *ver*, which encodes a KAS III homolog. Heterologous expression and promoter engineering successfully activated this BGC, affording eight non-acetate primed aromatic polyketides, verrucones A–H (**1**–**8**). These compounds feature three distinct skeletal types: isolated benzene ring, biphenyl, and anthraquinone. The successful activation of *ver* BGC underscores the utility of heterologous expression as a general strategy for targeted mining of chemically novel compounds from sequenced genomes. Among the isolates, compound **4** exhibited potent inhibitory activity against multiple PTP targets, providing a valuable lead for further drug discovery efforts targeting metabolic disorders such as diabetes and obesity. Our work not only enriches the structural diversity of aromatic polyketides through the incorporation of unusual starter units but also establishes a foundation for future pathway engineering to generate designer analogs with enhanced bioactivities.

## Figures and Tables

**Table 1:** The ^1^H (600 MHz) and ^13^C NMR (150 MHz) data of **1**–**3** in DMSO-*d*_6._

No.	1		2		3	
	*δ*_C_, Type	*δ*_H_, Mult. (*J* in Hz)	*δ*_C_, Type	*δ*_H_, Mult. (*J* in Hz)	*δ*_C_, Type	*δ*_H_, Mult. (*J* in Hz)
1	163.7, C		163.7, C		163.9, C	
2	88.4, CH	5.19, d (1.9)	88.4, CH	5.20, d (2.0)	88.5, CH	5.12, s
3	170.3, C		170.4, C		170.6, C	
4	100.8, CH	5.66, d (1.9)	100.8, CH	5.67, d (2.0)	100.9, CH	5.62, s
5	164.9, C		165.0, C		165.1, C	
6	36.8, CH_2_	3.57, s	36.6, CH_2_	3.64, s	36.9, CH_2_	3.56, s
7	137.9, C		134.0, C		138.3, C	
8	110.0, CH	6.14, d (2.2)	120.9, CH	6.71, d (7.6)	110.3, CH	6.14, d (2.3)
9	161.0, C		130.2, CH	7.19, t (7.9)	161.3, C	
10	101.7, CH	6.21, d (2.2)	114.8, CH	6.74, d (8.2)	101.8, CH	6.20, d (2.3)
11	161.0, C		155.1, C		161.5, C	
12	119.4, C		130.8, C		119.2, C	
13	199.8, C		199.7, C		200.1, C	
14	120.8, C		119.3, C		120.7, C	
15	157.0, C		159.7, C		156.6, C	
16	100.2, CH	6.09, d (2.0)	100.1, CH	6.06, d (2.2)	100.2, CH	6.08, d (2.1)
17	159.9, C		160.1, C		159.9, C	
18	104.3, CH	6.15, d (2.0)	105.2, CH	6.19, d (2.2)	104.2, CH	6.20, d (2.1)
1′	148.6, C		151.4, C		149.5, C	
2′	36.6, CH	2.48, m	36.0, CH	2.72, m	29.9, CH	2.76, m
3′	30.4, CH_2_	1.43, m	30.6, CH_2_	1.47, m	24.0, CH_3_	1.01, d (6.7)
		1.33, m		1.33, m		
4′	12.2, CH_3_	0.66, t (7.3)	12.2, CH_3_	0.68, t (7.4)	24.0, CH_3_	1.01, d (6.7)
5′	21.4, CH_3_	0.97, d (6.7)	21.5, CH_3_	0.98, d (6.7)		
3-OH		11.13, s		9.83, brs		
9-OH		10.13. s				
11-OH		11.61, s		11.6, s		
15-OH		9.90, s		10.48, s		
17-OH		9.61, s		9.70. s		

**Table 2:** The ^1^H (600 MHz) and ^13^C NMR (150 MHz) data of **4**–**5** in DMSO-*d*_6._

No.	4		5a		5b	
	*δ*_C_, Type	*δ*_H_, Mult. (*J* in Hz)	*δ*_C_, Type	*δ*_H_, Mult. (*J* in Hz)	*δ*_C_, Type	*δ*_H_, Mult. (*J* in Hz)
1	163.8, C		163.7, C		163.7, C	
2	89.4, CH	5.32, d (1.7)	88.4, CH	5.16, d (1.9)	88.4, CH	5.16, d (1.9)
3	170.9, C		170.2, C		170.2, C	
4	105.8, CH	6.21, d (1.7)	100.6, CH	5.55, d (1.9)	100.6, CH	5.54, d (1.9)
5	155.9, C		164.9, C		164.8, C	
6	127.3, C		37.1, CH_2_	3.53, d (9.9)	37.1, CH_2_	3.40, d (7.7)
				3.50, d (9.9)		3.38, d (7.7)
7	159.5, C		135.1, C		135.1, C	
8	113.7, C		120.1, CH	6.69, d (7.3)	120.0, CH	6.68, d (7.3)
9	189.0, C		128.5, CH	7.13, t (7.9)	128.4, CH	7.12, t (7.9)
10	108.8, C		114.0, CH	6.79, d (8.0)	113.9, CH	6.78, d (8.0)
11	164.6, C		145.5, C		145.4, C	
12	108.1, CH	6.58, d (2.0)	128.1, C		128.0, C	
13	166.4, C		137.9, C		137.7, C	
14	109.4, CH	7.13, d (2.0)	110.1, CH	5.89, d (2.3)	110.0, CH	5.88, d (2.3)
15	135.0, C		159.8, C		159.7, C	
16	181.1, C		101.8, CH	6.29, d (2.3)	101.8, CH	6.28, d (2.3)
17	133.8, C		158.8, C		158.7, C	
18	116.0, CH	7.64, s	118.8, C		118.6, C	
1′	155.5, C		209.4, C		209.2, C	
2′	38.0, CH	2.69, m	45.7, CH	2.55, m	45.9, CH	2.56, m
3′	29.6, CH_2_	1.62, m	25.7, CH_2_	1.49, m	25.4, CH_2_	1.31, m
				1.08, m		0.99, m
4′	11.9, CH_3_	0.74, t (7.3)	11.4, CH_3_	0.64, t (7.4)	11.2, CH_3_	0.45, t (7.4)
5′	21.2, CH_3_	1.21, d (6.8)	15.5, CH_3_	0.63, d (6.8)	15.9, CH_3_	0.82, d (6.8)
3-OH				11.59, brs		11.59, brs
11-OH				9.38, s		9.36, s
15-OH				9.86, brs		9.86, brs
17-OH				10.70, s		10.65, s

**Table 3:** The ^1^H (600 MHz) and ^13^C NMR (150 MHz) data of **6**–**8** in DMSO-*d*_6._

No.	6		7		8	
	*δ*_C_, Type	*δ*_H_, Mult. (*J* in Hz)	*δ*_C_, Type	*δ*_H_, Mult. (*J* in Hz)	*δ*_C_, Type	*δ*_H_, Mult. (*J* in Hz)
1	164.3, C		164.3, C		164.4, C	
2	92.8, C		92.7, C		93.6, C	
3	177.6, C		177.7, C		176.9, C	
4	108.9, CH	5.23, s	108.6, CH	5.21, s	112.2, CH	5.65, s
5	160.1, C		160.2, C		152.9, C	
6	36.6, CH_2_	3.31, s	36.4, CH_2_	3.41, s	128.9, C	
7	139.7, C		135.1, C		159.5, C	
8	109.5, CH	6.07, d (2.2)	120.1, CH	6.66, d (7.9)	113.9, C	
9	161.8, C		129.8, CH	7.15, t (7.9)	187.2, C	
10	101.2, CH	6.15, d (1.8)	114.3, CH	6.70, d (7.9)	107.2, C	
11	161.6, C		154.9, C		164.4, C	
12	118.6, C		130.8, C		108.0, CH	6.38, s
13	200.0, C		199.7, C		164.9, C	
14	120.9, C		118.6, C		111.7, CH	7.02, s
15	157.1, C		160.7, C		134.9, C	
16	100.3, CH	6.11, d (2.0)	100.2, CH	6.05, d (2.0)	181.8, C	
17	160.1, C		161.8, C		133.4, C	
18	104.4, CH	6.16, d (2.0)	105.5, CH	6.17, d (2.0)	115.7, CH	7.59, s
1′	148.5, C		151.7, C		154.5, C	
2′	36.6, CH	2.50, m *^a^*	35.8, CH	2.72, m *^a^*	37.7, CH	2.78, m *^a^*
3′	30.4, CH_2_	1.43, m	30.7, CH_2_	1.44, m *^a^*	29.6, CH_2_	1.61, m *^a^*
		1.34, m		1.30, m		
4′	12.2, CH_3_	0.67, t (7.2)	12.2, CH_3_	0.64, t (7.3)	12.1, CH_3_	0.73, t (7.3)
5′	21.5, CH_3_	0.98, d (6.7)	21.6, CH_3_	0.95, d (6.6)	21.5, CH_3_	1.19, d (6.8)
1″	53.7, CH	3.98, dt (2.8, 12.0)	53.7, CH	3.98, dt (2.3, 11.9)	53.7, CH	4.10, d (11.3)
2″	27.5, CH_2_	1.93, m	27.5, CH_2_	1.93, m	27.5, CH_2_	2.05, m
		1.58, m		1.58, m		1.71, m *^a^*
3″	22.5, CH_2_	1.72, m	22.5, CH_2_	1.73, m	22.5, CH_2_	1.78, m
		1.45, m *^a^*		1.44, m *^a^*		1.50, m
4″	22.1, CH_2_	1.68, m	22.1, CH_2_	1.68, m	22.2, CH_2_	1.71, m *^a^*
		1.52, m		1.53, m		1.61, m *^a^*
5″	44.2, CH_2_	3.23, m	44.1, CH_2_	3.24, m	44.2, CH_2_	3.27, d (11.6)
		2.74, m		2.72, m *^a^*		2.78, m *^a^*

*^a^*: overlapped.

**Table 4:** PTP inhibitory activities of **1**–**8**.

Compound	IC_50_ (μM)			
	PTP1B	SHP1	SHP2	TCPTP
**1**	3.65 ± 0.28	6.79 ± 0.39	15.22 ± 0.86	2.34 ± 0.37
**2**	5.35 ± 0.33	24.82 ± 0.44	>50	6.08 ± 0.45
**3**	8.23 ± 0.39	/	>50	7.51 ± 0.58
**4**	1.84 ± 0.26	1.84 ± 0.38	15.37 ± 1.01	1.84 ± 0.33
**5**	12.38 ± 1.09	/	/	10.24 ± 1.37
**6**	>50	/	/	>50
**7**	>50	/	/	>50
**8**	>50	/	/	>50
**Na_3_VO_4_ *^a^***	1.02 ± 0.30	3.40 ± 0.63	4.82 ± 0.84	2.71 ± 0.48

*^a^* Na_3_VO_4_ was used as a positive control. /: Not detected.

## Data Availability

The data presented in this study are available in the article and Supplementary Materials.

## Supplementary Materials

The following supporting information can be downloaded at: https://www.mdpi.com/article/10.3390/microorganisms14081669/s1. Table S1: The media used in this study; Table S2: Strains and plasmids used in this study; Table S3: Primers used in this study; Table S4: Deduced functions of ORFs in the *ver* biosynthetic gene cluster; Table S5: Cytotoxicity activity of verrucone C (**3**); Figure S1: Comparison of *ver* with homologous gene clusters; Figure S2: Construction of expression vector of the *ver* gene cluster; Figure S3: Construction of plasmid for the insertion of *kasOp** and the inactivation of *verA3*–*A5*; Figure S4: The HRMS data for verrucone A–H (**1**–**8**); Figure S5: The UV absorbance spectra of verrucones A–H (**1**–**8**); Figures S6–S51: NMR spectra of verrucones A–H (**1**–**8**).
