# Supplementary material for: Verrucones A–H, Non-Acetate Starter Aromatic Polyketides Discovered by Heterologous Expression of a Type II PKS Gene Cluster
Source: Microorganisms. 2026 Jul 30;14(8):1669. doi: 10.3390/microorganisms14081669 (PMC13515585; doi:10.3390/microorganisms14081669)
Supplement: Supplementary file 1 [file microorganisms-14-01669-s001.zip › microorganisms-4436254-supplementary.pdf]

Supplementary Materials

Verrucones A−H, Non-acetate Starter Aromatic Polyketides Discovered by Heterologous Expression of a Type II PKS Gene Cluster

Xingkun Hao[1,†], Ming Yang[1,†], Ping Yan[1], Qiyao Shen[1], Yang Liu[1], Youming Zhang[1,*], Xiaoying Bian [1,*], Haibo Zhou[1,*]

[1]Helmholtz International Lab for Anti-infectives, Shandong University-Helmholtz Institute of Biotechnology, State Key Laboratory of Microbial Technology, Shandong University, Qingdao, Shandong 266237, China

* Youming Zhang, Email: zhangyouming@sdu.edu.cn

Xiaoying Bian, Email: bianxiaoying@sdu.edu.cn

Haibo Zhou, Email: haibozhou@sdu.edu.cn

† These authors contributed equally to this work.

Contents

Table S1. The media used in this study 4

Table S2. Strains and plasmids used in this study 4

Table S3. Primers used in this study 5

Table S4. Deduced functions of ORFs in the *ver* biosynthetic gene cluster 5

Table S5. Cytotoxicity activity of verrucone C (**3**) 7

Figure S1. Comparison of *ver* with homologous gene clusters. 8

Figure S2. Construction of expression vector of the *ver* gene cluster. 8

Figure S3. Construction of plasmid for the insertion of *kasO*p* and the inactivation of *verA3−A5*. 9

Figure S4. The HRMS data for verrucones A−H (**1−8**). 9

Figure S5. The UV absorbance spectra of verrucones A−H (**1−8**) 10

Figure S6. ^{1}H NMR spectrum (600 MHz) of verrucone A (**1**) in DMSO-d_6. 11

Figure S7. ^{13}C NMR spectrum (150 MHz) of verrucone A (**1**) in DMSO-d_6. 11

Figure S8. DEPT spectrum (150 MHz) of verrucone A (**1**) in DMSO-d_6. 12

Figure S9. HSQC spectrum (150 MHz/600 MHz) of verrucone A (**1**) in DMSO-d_6. 12

Figure S10. ^{1}H-^{1}H COSY spectrum (600 MHz) of verrucone A (**1**) in DMSO-d_6. 13

Figure S11. HMBC spectrum (150 MHz/600 MHz) of verrucone A (**1**) in DMSO-d_6. 13

Figure S12. ^{1}H NMR spectrum (600 MHz) of verrucone B (**2**) in DMSO-d_6. 14

Figure S13. ^{13}C NMR spectrum (150 MHz) of verrucone B (**2**) in DMSO-d_6. 14

Figure S14. DEPT spectrum (150 MHz) of verrucone B (**2**) in DMSO-d_6. 15

Figure S15. HSQC spectrum (150 MHz/600 MHz) of verrucone B (**2**) in DMSO-d_6. 15

Figure S16. ^{1}H-^{1}H COSY spectrum (600 MHz) of verrucone B (**2**) in DMSO-d_6. 16

Figure S17. HMBC spectrum (150 MHz/600 MHz) of verrucone B (**2**) in DMSO-d_6. 16

Figure S18. ^{1}H NMR spectrum (600 MHz) of verrucone C (**3**) in DMSO-d_6. 17

Figure S19. ^{13}C NMR spectrum (150 MHz) of verrucone C (**3**) in DMSO-d_6. 17

Figure S20. HSQC spectrum (150 MHz/600 MHz) of verrucone C (**3**) in DMSO-d_6. 18

Figure S21. ^{1}H-^{1}H COSY spectrum (600 MHz) of verrucone C (**3**) in DMSO-d_6. 18

Figure S22. HMBC spectrum (150 MHz/600 MHz) of verrucone C (**3**) in DMSO-d_6. 19

Figure S23. ^{1}H NMR spectrum (600 MHz) of verrucone D (**4**) in DMSO-d_6. 19

Figure S24. ^{13}C NMR spectrum (150 MHz) of verrucone D (**4**) in DMSO-d_6. 20

Figure S25. DEPT spectrum (150 MHz) of verrucone D (**4**) in DMSO-d_6. 20
Figure S26. HSQC spectrum (150MHz/600 MHz) of verrucone D (**4**) in DMSO-d_6......... 21
Figure S27. ^{1}H-^{1}H COSY spectrum (600 MHz) of verrucone D (**4**) in DMSO-d_6. 21
Figure S28. HMBC spectrum (150MHz/600 MHz) of verrucone D (**4**) in DMSO-d_6. 22
Figure S29. ^{1}H NMR spectrum (600 MHz) of verrucone E (**5**) in DMSO-d_6.................... 22
Figure S30. ^{13}C NMR spectrum (150 MHz) of verrucone E (**5**) in DMSO-d_6................... 23
Figure S31. HSQC spectrum (150 MHz/600 MHz) of verrucone E (**5**) in DMSO-d_6. 23
Figure S32. ^{1}H-^{1}H COSY spectrum (600 MHz) of verrucone E (**5**) in DMSO-d_6............. 24
Figure S33. HMBC spectrum (150 MHz/600 MHz) of verrucone E (**5**) in DMSO-d_6....... 24
Figure S34. ^{1}H NMR spectrum (600 MHz) of verrucone F (**6**) in DMSO-d_6. 25
Figure S35. ^{13}C NMR spectrum (150 MHz) of verrucone F (**6**) in DMSO-d_6. 25
Figure S36. DEPT spectrum (150 MHz) of verrucone F (**6**) in DMSO-d_6. 26
Figure S37. HSQC spectrum (150 MHz/600 MHz) of verrucone F (**6**) in DMSO-d_6. 26
Figure S38. ^{1}H-^{1}H COSY spectrum (600 MHz) of verrucone F (**6**) in DMSO-d_6............. 27
Figure S39. HMBC spectrum (150 MHz/600 MHz) of verrucone F (**6**) in DMSO-d_6. 27
Figure S40. ^{1}H NMR spectrum (600 MHz) of verrucone G (**7**) in DMSO-d_6. 28
Figure S41. ^{13}C NMR spectrum (150 MHz) of verrucone G (**7**) in DMSO-d_6. 28
Figure S42. DEPT spectrum (150 MHz) of verrucone G (**7**) in DMSO-d_6. 29
Figure S43. HSQC spectrum (150 MHz/600 MHz) of verrucone G (**7**) in DMSO-d_6........ 29
Figure S44. ^{1}H-^{1}H COSY spectrum (600 MHz) of verrucone G (**7**) in DMSO-d_6. 30
Figure S45. HMBC spectrum (150 MHz/600 MHz) of verrucone G (**7**) in DMSO-d_6. 30
Figure S46. ^{1}H MNR spectrum (600 MHz) of verrucone H (**8**) in DMSO-d_6. 31
Figure S47. ^{13}C MNR spectrum (150 MHz) of verrucone H (**8**) in DMSO-d_6. 31
Figure S48. DEPT spectrum (150 MHz) of verrucone H (**8**) in DMSO-d_6. 32
Figure S49. HSQC spectrum (150 MHz/600 MHz) of verrucone H (**8**) in DMSO-d_6. 32
Figure S50. ^{1}H-^{1}H COSY spectrum (600 MHz) of verrucone H (**8**) in DMSO-d_6. 33
Figure S51. HMBC spectrum (150 MHz/600 MHz) of verrucone H (**8**) in DMSO-d_6. 33

Supplementary Tables:

Table S1. The media used in this study

Media	Compositions
ISP2	0.4% yeast extract, 1% malt extract, 0.4% glucose, 2% agar, seawater, pH 7.3
YEME	5% soluble starch, 0.3% yeast extract, 0.5% peptone, 1% glucose, seawater, pH 7.0
YM	1% soluble starch, 0.5% yeast extract, 0.5% KNO_3, 0.5% NaCl

The seawater, derived from the natural waters of Aoshan Bay, has a salinity of about 3.1%.

Table S2. Strains and plasmids used in this study

Strains	Characteristics	Source
Streptomyces sp. S42	Wild type	Our lab
E. coli GB2005	DH10B, *fhu*A:: IS2, Δ*rec*ET, Δ*ybc*C	Our lab
E. coli GB05-dir	GB2005, *ybc*C::P_{BAD}-*ETγ*A, used to mediate LLHR	Our lab
E. coli GB08-red	GB2005, *ybc*C::P_{BAD}-*γβα*A, used to mediate LCHR	Our lab
E. coli GBred-gyrA462	CcdB-resistant strain used to mediate LCHR	Our lab
E. coli ET12567/pUZ8002	Conjugative transfer donor bacteria	Our lab
S. albus J1074	Heterologous expression host bacteria	Our lab
J1074-*ver*	*S. albus* J1074 contains plasmid pBAC-*ver*	This study
J1074-*ver*-*kasO*p*	*S. albus* J1074 contains plasmid pBAC-*ver*-*kasO*p*	This study
J1074-*ver*-ΔPKS	*S. albus* J1074 contains plasmid pBAC-*ver*-ΔPKS	This study
**Plasmids**	**Characteristics**	**Source**
pBeloBAC11	PCR template for direct cloning vector	Our lab
pSC101-BAD-EtgA-tet	Temperature-sensitive plasmid for Red/ET recombination	Our lab
pR6K-oriT-phiC31	PCR template of the *oriT-attP-phiC31* cassette	Our lab
p15A-amp-ccdB	PCR template of amp-ccdB	Our lab
p15A-*ermE**p-hyg-*kasO*p*	PCR template of the *ermE*p-hyg-kasOp** cassette	Our lab
pBAC-*ver*	pBeloBAC11 harboring *ver* gene cluster	This study
pBAC-*ver*-apra-phiC31	The *oriT-attP-phiC31* cassette was inserted into pBAC-*ver* for targeting in heterologous host's genomes	This study
pBAC-*ver*-*kasO*p*	The *ermE*p-hyg-kasOp** cassette was inserted into *ver* cluster before *verA3*	This study
pBAC-*ver*-ΔPKS	*verA3-A5* genes were deleted	This study

Table S3. Primers used in this study

Primer name	Sequence[a] (5′-3′)	Application
YM-*ver*-HAF	ccgtcggtcgcggagatccgcgagaagttccagaagcgccgcgcggccggcgcgggccgtctcgaccaggcgctgcgcCACGCTGATAGTCTGATCGACA	pBAC-*ver*
YM-*ver*-HAR	ccgggccgtcttccgtggtcagccgccccttggcgcccatgcacgggacaccggggagcacaaaaagaaccaaaggtgAGGGCACCAATAACTGCCTTAA	
ver-apra-phiC31-HAF	gtccctgttgataccgggaagccctgggccaacttttggcgGGTTCATGTGCAGCTCCA	pBAC-*ver*-apra-phiC31
ver-apra-phiC31-HAR	agggcaccaataactgccttaaaaaaattacgccccgccctgCTACGCCGCTACGTCTT	
ver-*kasO*p*-HAF	ggcaacgtcccgctgtagtgcccaaaggccctcggaggcggtggccgccCTACGATGTATCAGGCGCC	pBAC-*ver*-*kasO*p*
ver-*kasO*p*-HAR	cagacctgtgatcacgactctgcgtaccaccgcttactccccttcgacCCATGGATCCTACACCAGA	
ver-ΔPKS-amp-HAF	cgaaggggaggtaagcggtggtacgcagagtcgtgatcacaggtctgTTAATTAATTCAAATATGTATCCGCTCATG	pBAC-*ver*-ΔPKS
ver-ΔPKS-amp-HAR	gcgcaggacgagcgccgcgttgaagccgccgtggccgcgggccagcattaattaaGTATTCACGAACGAAAATCGC	

[a] The homology arms are in lower case.

Table S4. Deduced functions of ORFs in the *ver* biosynthetic gene cluster

ORF	size (aa)	proposed function	identity	protein ID
*verT*1	152	nuclear transport factor 2 family protein	70%	TxnO10, AKT74297.1, *Streptomyces bottropensis* DO-45
verB	40	hypothetical protein	95%	WP_277402560.1, *Streptomyces verrucosisporus*
verH1	324	MBL fold metallo-hydrolase	73%	LldC1, QDQ37880.1, *Streptomyces vinaceusdrappus*
verO1	172	flavin reductase family protein	62%	TxnO10, AKT74294.1, *Streptomyces bottropensis* DO-45
verO2	261	SDR family NAD(P)-dependent oxidoreductase	76%	TxnC2, AKT74293.1, *Streptomyces bottropensis* DO-45
verT2	165	SRPBCC family protein	78%	LldC3, QDQ37883.1, *Streptomyces vinaceusdrappus*
verH2	380	hydrolase	74%	LldO5, QDQ37884.1, *Streptomyces vinaceusdrappus*
verO3	434	cytochrome P450	81%	TxnO6, AKT74289.1, *Streptomyces bottropensis* DO-45
verO4	255	LLM class flavin-dependent oxidoreductase	96%	WP_206350148.1, *Streptomyces verrucosisporus*
verD	183	hypothetical protein	95%	WP_241713525.1, *Streptomyces sp.* CNQ085
verO5	452	NAD(P)/FAD-dependent oxidoreductase	60%	TxnO3, AKT74277.1, *Streptomyces bottropensis* DO-45
verE	107	2Fe-2S iron-sulfur cluster-binding protein	80%	TxnO4, AKT74278.1, *Streptomyces bottropensis* DO-45
verT3	502	MFS transporter	93%	WP_101255209.1, *Streptomyces barkulensis*
verH3	502	alpha/beta fold hydrolase	67%	TxnH1, AKT74279.1, *Streptomyces bottropensis* DO-45
verO6	252	SDR family NAD(P)-dependent	69%	TxnP4, AKT74271.1,

		oxidoreductase		*Streptomyces bottropensis* DO-45
verF	370	winged helix DNA-binding domain-containing protein	51%	TxnU2, AKT74276.1, *Streptomyces bottropensis* DO-45
verO7	345	MDR family NADP-dependent oxidoreductase	56%	TxnO1, AKT74273.1, *Streptomyces bottropensis* DO-45
verO8	424	cytochrome P450	53%	TxnO6, AKT74289.1, *Streptomyces bottropensis* DO-45
verH4	373	epoxide hydrolase family protein	74%	TxnH2, AKT74281.1, *Streptomyces bottropensis* DO-45
verO9	407	cytochrome P450	66%	TxnO5, AKT74280.1, *Streptomyces bottropensis* DO-45
verG	403	CaiB/BaiF CoA transferase family protein	66%	TxnP3, AKT74270.1, *Streptomyces bottropensis* DO-45
verT4	559	MFS transporter	51%	TxnRg2, AKT74287.1, *Streptomyces bottropensis* DO-45
verI	551	citramalate synthase	67%	TxnP2, AKT74269.1, *Streptomyces bottropensis* DO-45
verK	345	ketoacyl-ACP synthase III family protein	73%	TxnA5, AKT74268.1, *Streptomyces bottropensis* DO-45
verA1	343	aromatase/cyclase	59%	TxnC1, AKT74267.1, *Streptomyces bottropensis* DO-45
verO10	210	antibiotic biosynthesis monooxygenase family protein	90%	WP_410537349.1, *Streptomyces* sp. KL2
verJ	81	acyl-CoA carboxylase subunit epsilon	98%	WP_241713502.1. *Streptomyces* sp. CNQ085
verL	642	fatty acyl-AMP ligase	52%	TxnP1, AKT74266.1, *Streptomyces bottropensis* DO-45
verO11	575	acyl-CoA dehydrogenase family protein	90%	WP_093657602.1, *Streptomyces radiopugnans*
verC1	413	acyltransferase domain-containing protein	95%	WP_093852284.1, *Streptomyces*
verA2	299	4'-phosphopantetheinyl transferase family protein	99%	WP_241713495.1, *Streptomyces* sp. CNQ085
verR1	204	response regulator transcription factor	70%	TxnRg4, AKT74265.1, *Streptomyces bottropensis* DO-45
verR2	420	sensor histidine kinase	91%	WP_279950811.1, *Streptomyces chitinivorans*
verM	786	TOMM precursor leader peptide-binding protein	92%	WP_436991928.1, *Streptomyces* sp. enrichment culture
verN	122	hypothetical protein	94%	WP_093852281.1, *Streptomyces*
verR3	264	AfsR/SARP family transcriptional regulator	76%	TxnRg2, AKT74263.1, *Streptomyces bottropensis* DO-45
verP	545	acyl-CoA carboxylase subunit beta	96%	WP_410537337.1, *Streptomyces sp.* KL2
verA3	422	T2PKS: beta-ketoacyl synthase	88%	TxnA1, AKT74262.1, *Streptomyces bottropensis* DO-45
verA4	424	T2PKS: ketosynthase chain-length factor	78%	TxnA2, AKT74261.1, *Streptomyces bottropensis* DO-45
verA5	91	acyl carrier protein	75%	TxnA3, AKT74260.1, *Streptomyces bottropensis* DO-45
verC2	619	acyltransferase domain-containing protein	56%	TxnA4, AKT74259.1, *Streptomyces bottropensis* DO-45
verQ	54	hypothetical protein	67%	WP_206350091.1, *Streptomyces verrucosisporus*
verS	217	SRPBCC family protein	86%	WP_241713481.1, *Streptomyces* sp. CNQ085

Table S5. Cytotoxicity activity of verrucone C (**3**)

compound	IC_{50} (μM) ±SD					
	MCF-7	HepG-2	HT-29	293T	HL-60	Jurkat
**3**	16.387±1.320	> 20	> 20	> 20	> 20	> 20
Dox[a]	2.709±0.687	0.689±0.092	0.507±0.067	1.576±0.246	0.177±0.410	0.147±0.032

[a]: Doxorubicin, positive control.

MCF-7: human breast cancer cell line, HepG2: human liver cancer cell line, HT29: human colorectal adenocarcinoma cell line, HL60: human promyelocytic leukemia cell line, Jurkat: Human acute T-cell leukemia cell line, 293T: Human embryonic kidney cell line; Dox: Doxorubicin.

Supplementary Figures:

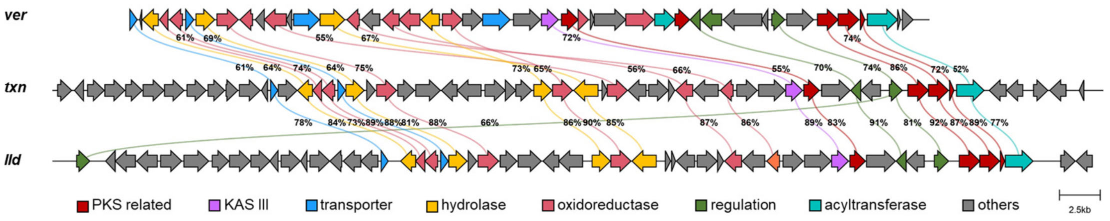

Figure S1. Comparison of *ver* with homologous gene clusters. The values on the lines represent the identities between homologous genes. Legend: *ver*, verrucone; *txn*, trioxacarcin A; *lld*, LL-D49194α1.

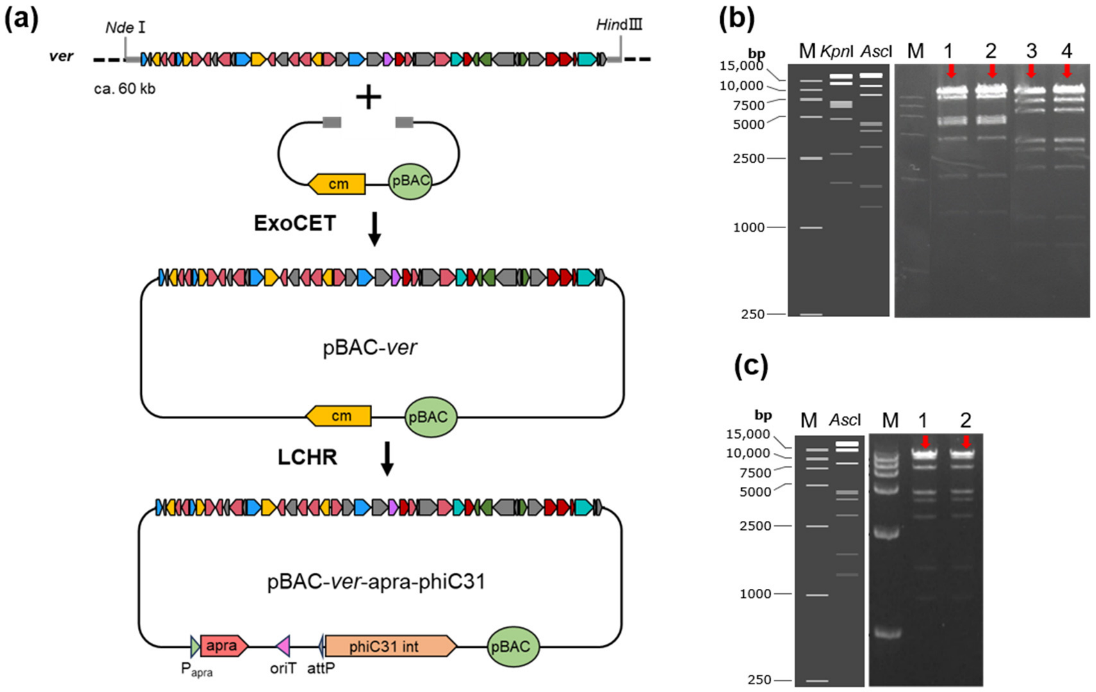

Figure S2. Construction of expression vector of the *ver* gene cluster. (a) Schematic representation of the direct cloning and engineering of the *ver* gene cluster by ExoCET and LCHR, respectively. The dotted box shows the real size of *ver* gene cluster; (b) Restriction endonuclease analysis of pBAC-*ver* using *Kpn*I (Lane 1-2) and *Asc*I (Lane 3-4). (c) Restriction endonuclease analysis of pBAC-*ver*-apra-phiC31 (Lane 1-2) using *Asc*I. Correct clones are indicated with red arrows. Lane M: 15 kb DNA ladder. The corresponding simulated gel image obtained by SnapGene 3.2 is shown on the left panel.

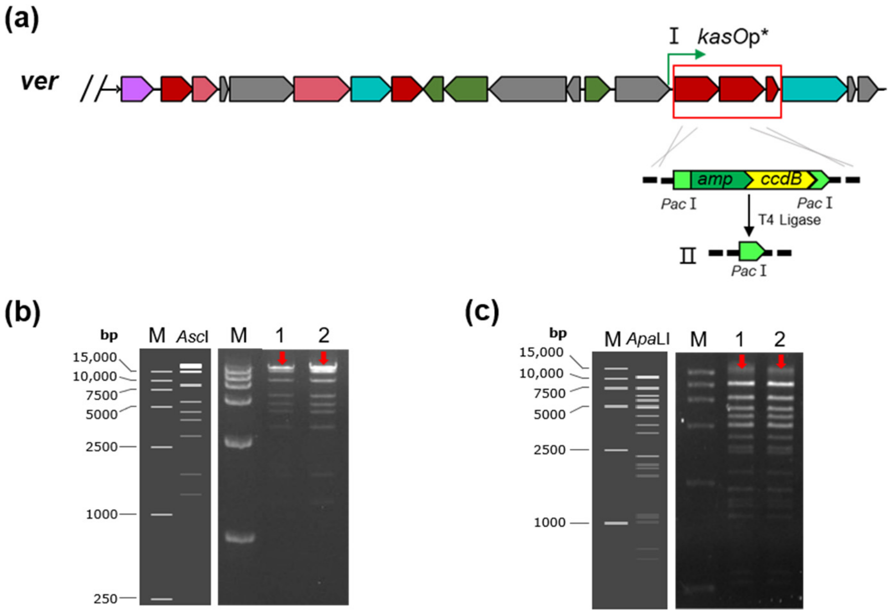

Figure S3. Construction of plasmid for the insertion of *kasO*p* and the inactivation of *verA3−A5*. (a) Schematic representation of the insertion of *kasO*p* and inactivation of *verA3−A5*; (b) Restriction endonuclease analysis of pBAC-*ver*-*kasO*p* (Lane 1-2) using *Asc*I. (c) Restriction endonuclease analysis of pBAC-*ver*-ΔPKS (Lane 1-2) using *Apa*LI. Correct clones are indicated with red arrows. Lane M: 15 kb DNA ladder. The corresponding simulated gel image obtained by SnapGene 3.2 is shown on the left panel.

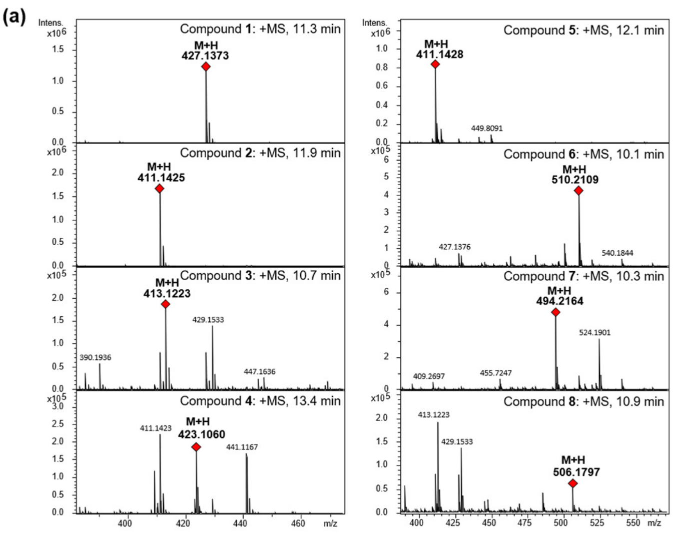

(b)

Compd.	molecular formulae	Calculated mass	Observed mass	Exact mass error (ppm)
1	$C_{23}H_{23}O_8$	427.1387	427.1373	-3.2776
2	$C_{23}H_{23}O_7$	411.1438	411.1425	-3.1619
3	$C_{23}H_{21}O_8$	413.1231	413.1223	-1.9365
4	$C_{23}H_{19}O_8$	423.1074	423.1060	-3.3088
5	$C_{23}H_{23}O_7$	411.1438	411.1428	-2.4322
6	$C_{28}H_{32}NO_8$	510.2122	510.2109	-2.5479
7	$C_{28}H_{32}NO_7$	494.2173	494.2164	-1.8211
8	$C_{28}H_{28}NO_8$	506.1809	506.1797	-2.3707

Figure S4. The HRMS data for verrucones A−H (**1**−**8**). (a) HRESIMS spectra of verrucones A−H (**1**−**8**). (b) Detailed mass for compounds **1**−**8**.

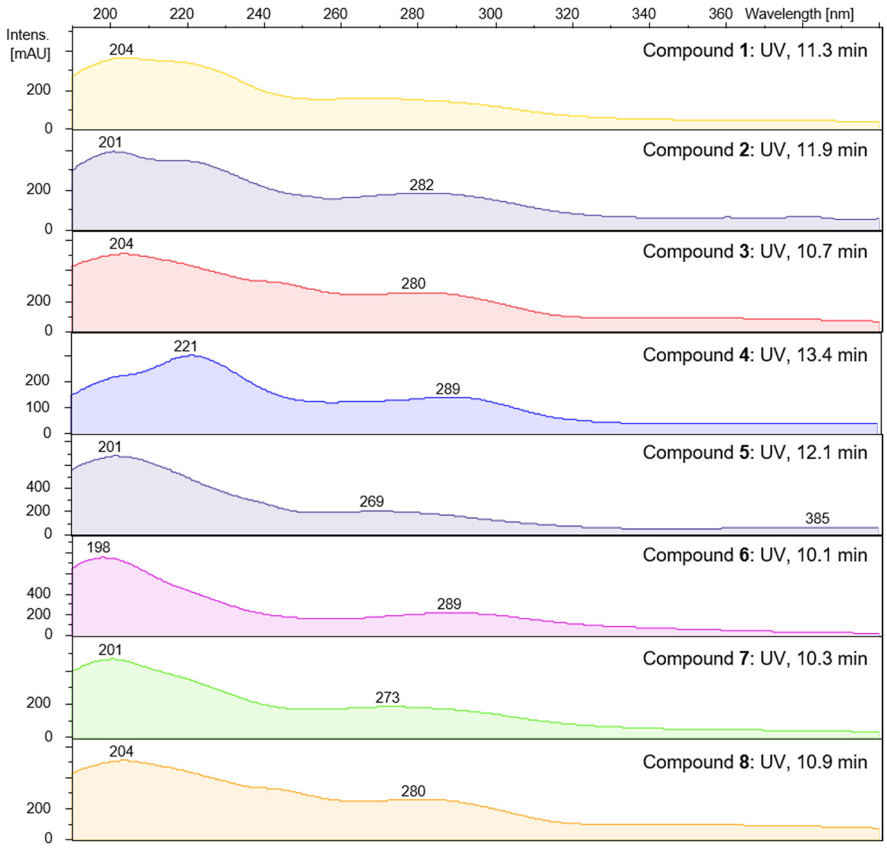

Figure S5. The UV absorbance spectra of verrucones A−H (**1**−**8**).

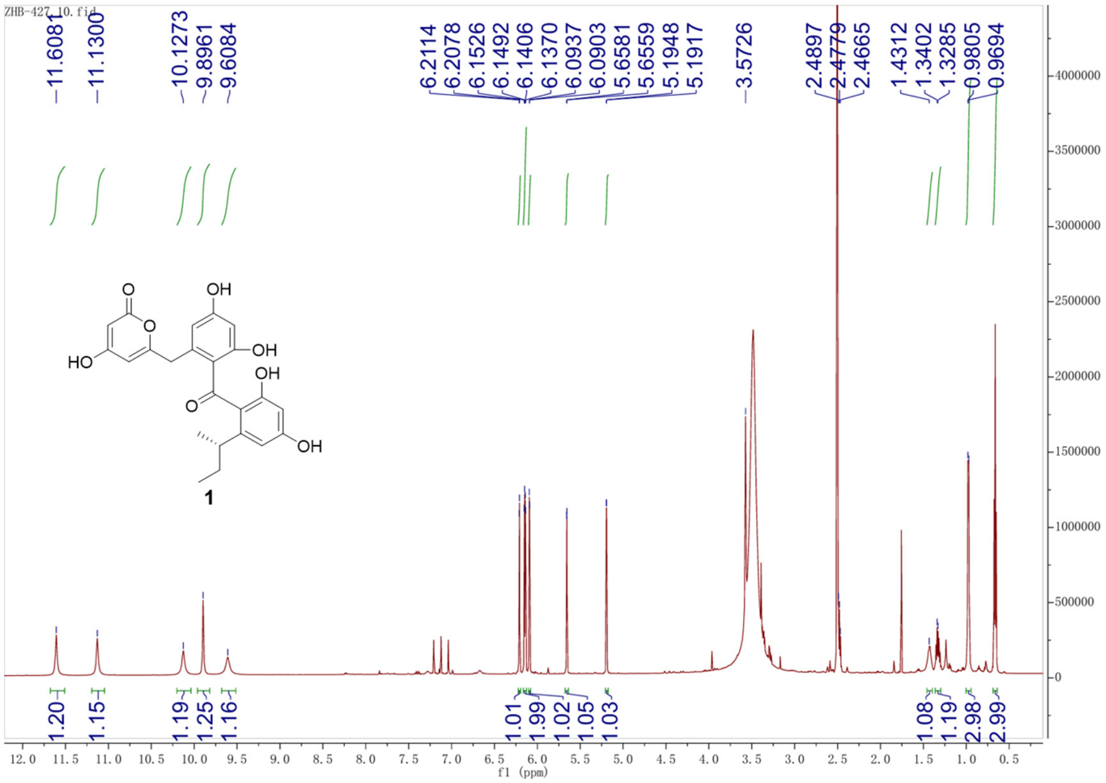

Figure S6. ^{1}H NMR spectrum (600 MHz) of verrucone A (**1**) in DMSO-d_6.

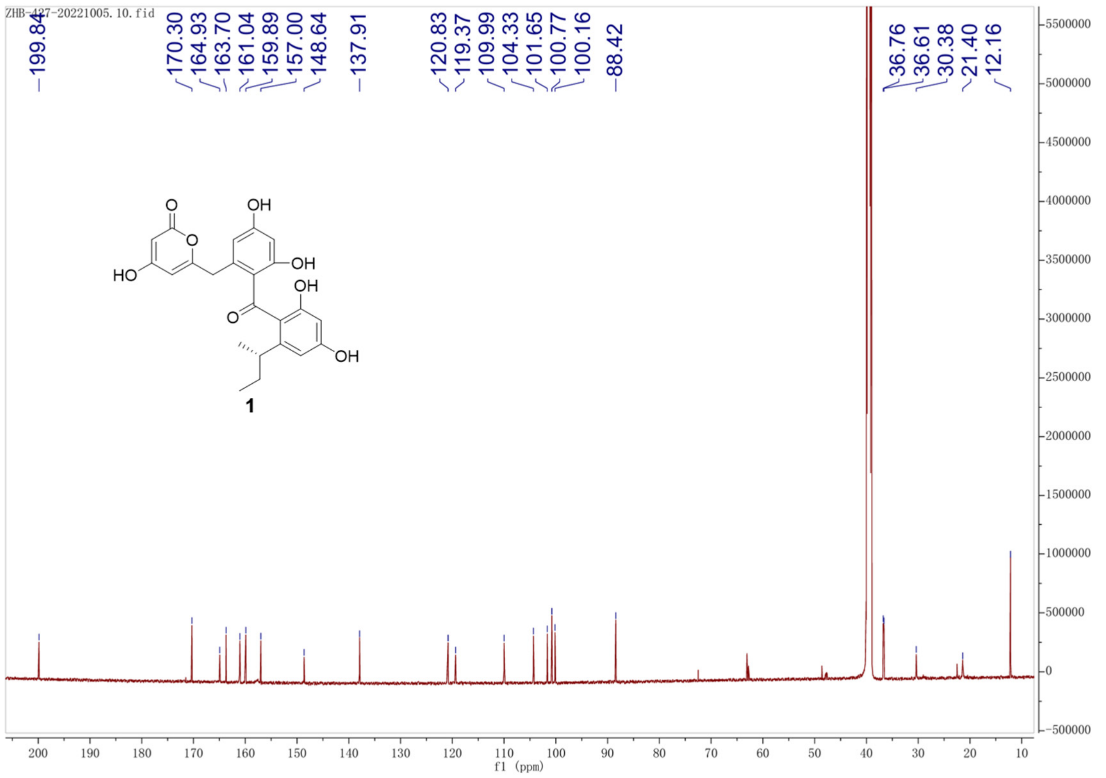

Figure S7. ^{13}C NMR spectrum (150 MHz) of verrucone A (**1**) in DMSO-d_6.

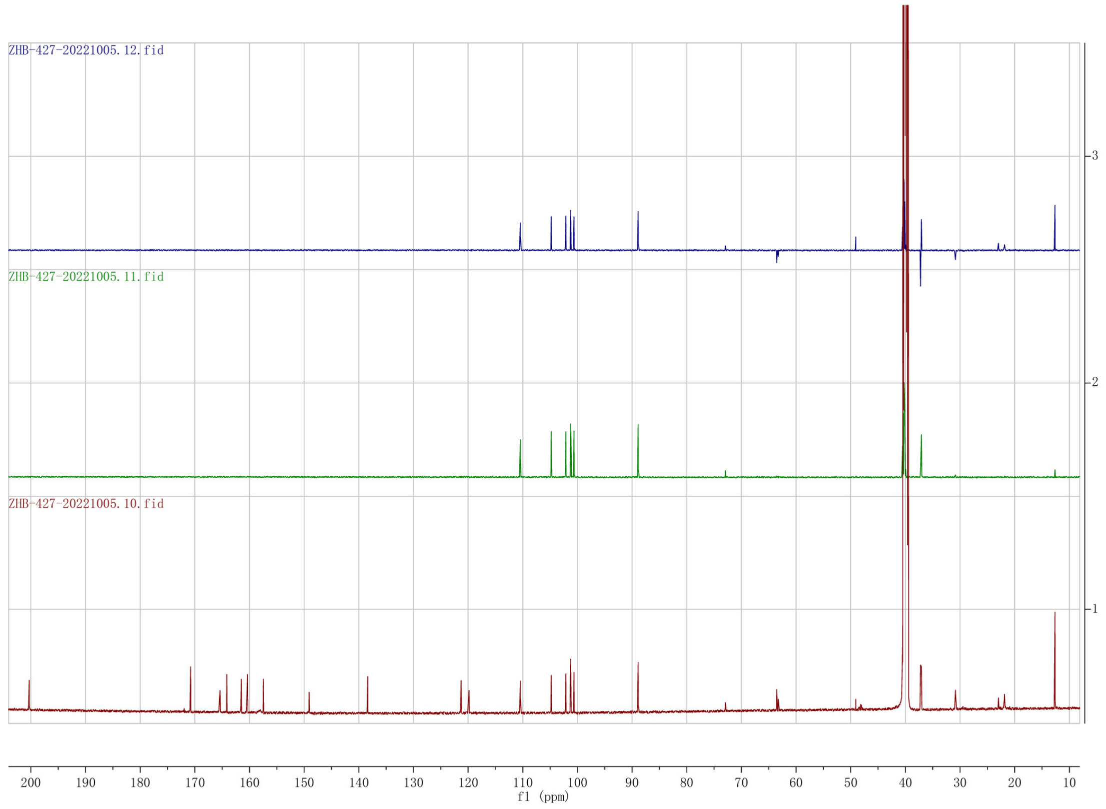

Figure S8. DEPT spectrum (150 MHz) of verrucone A (**1**) in DMSO-d_6.

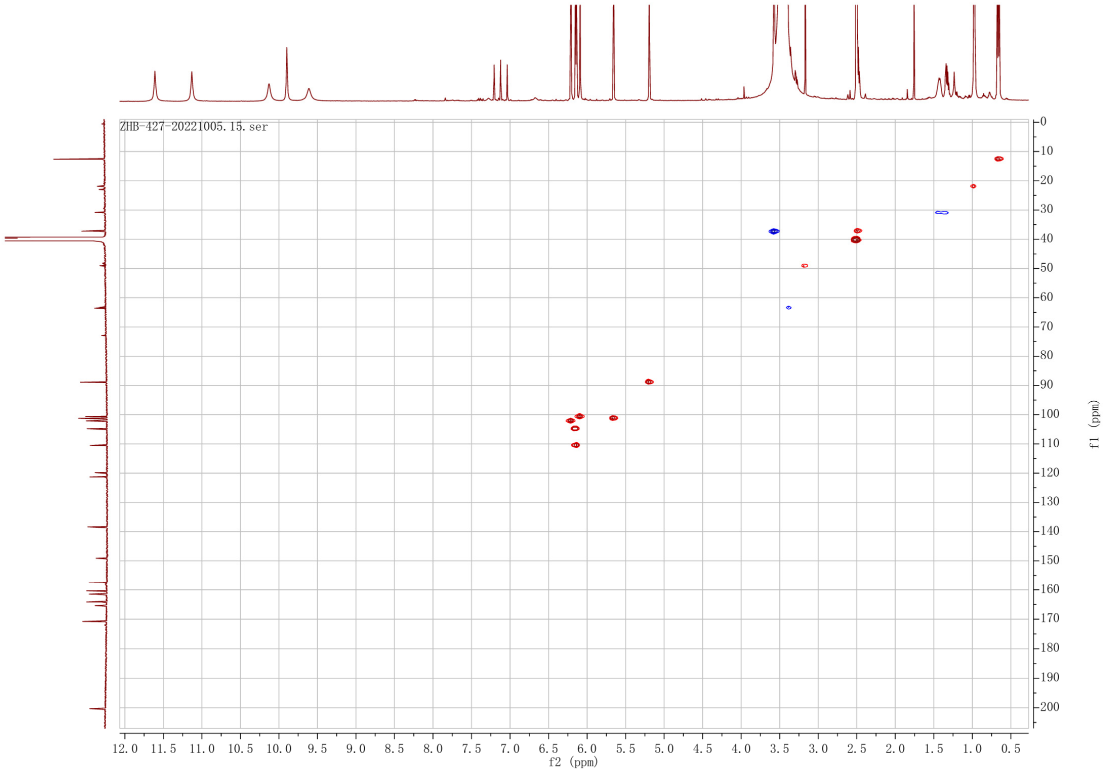

Figure S9. HSQC spectrum (150 MHz/600 MHz) of verrucone A (**1**) in DMSO-d_6.

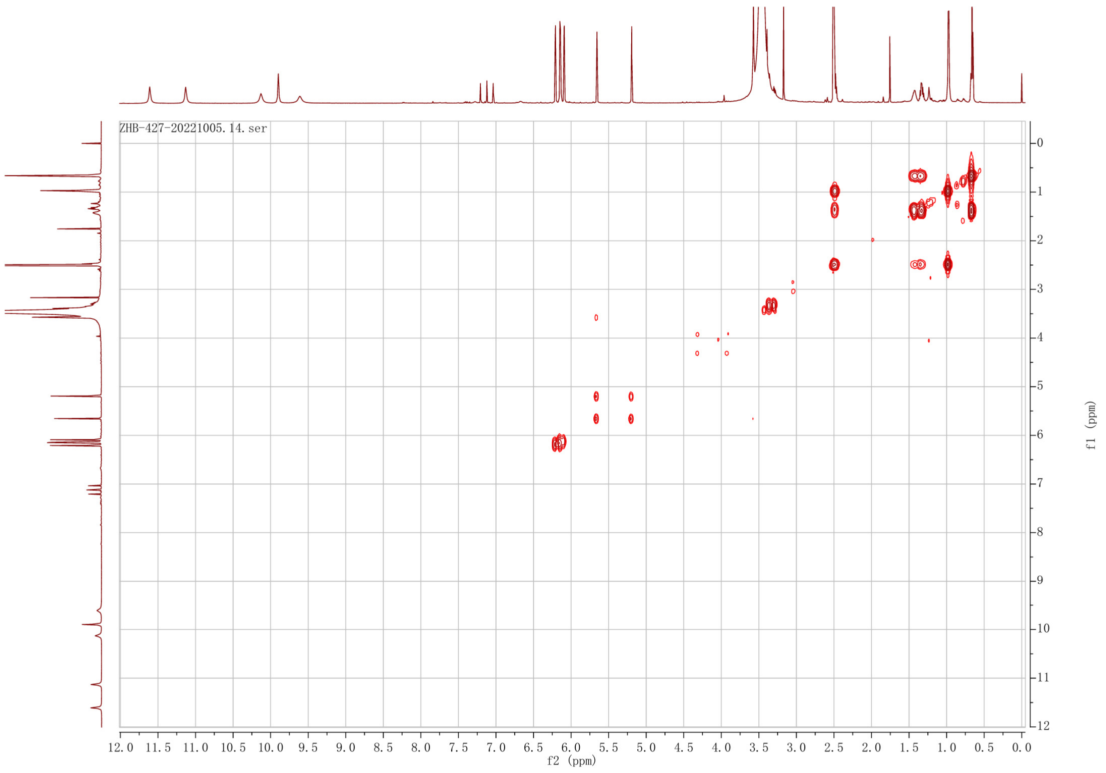

Figure S10. ^{1}H-^{1}H COSY spectrum (600 MHz) of verrucone A (**1**) in DMSO-d_6.

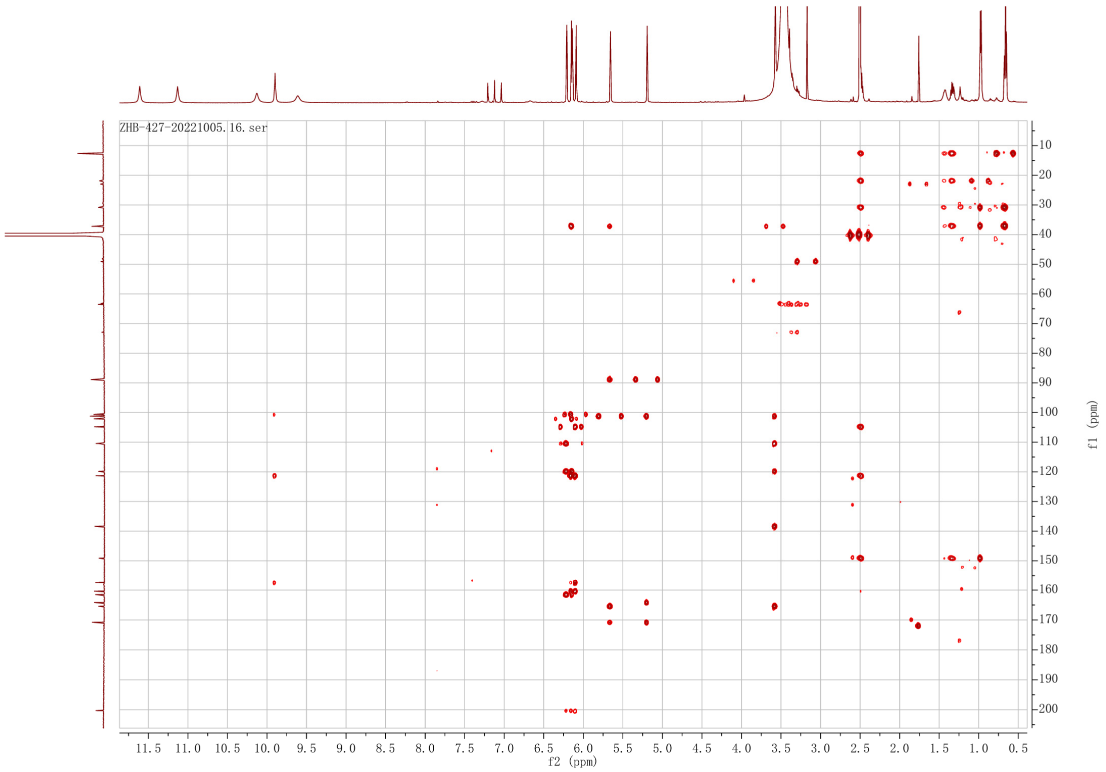

Figure S11. HMBC spectrum (150 MHz/600 MHz) of verrucone A (**1**) in DMSO-d_6.

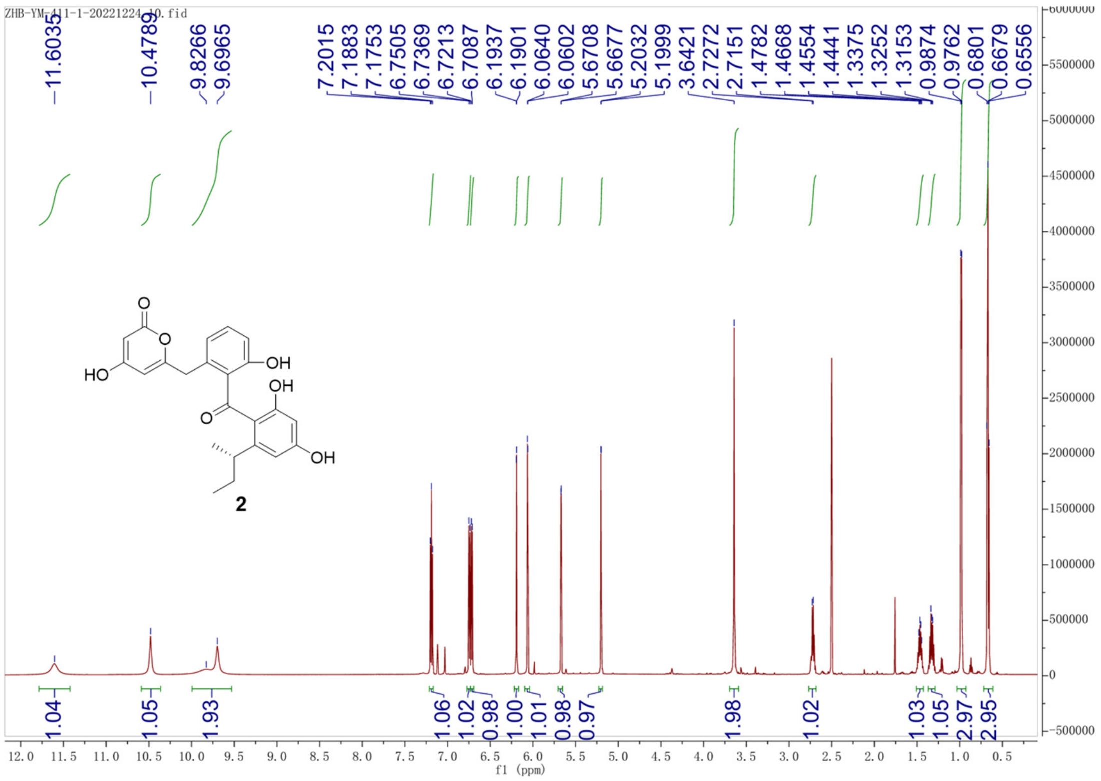

Figure S12. ^{1}H NMR spectrum (600 MHz) of verrucone B (**2**) in DMSO-d_6.

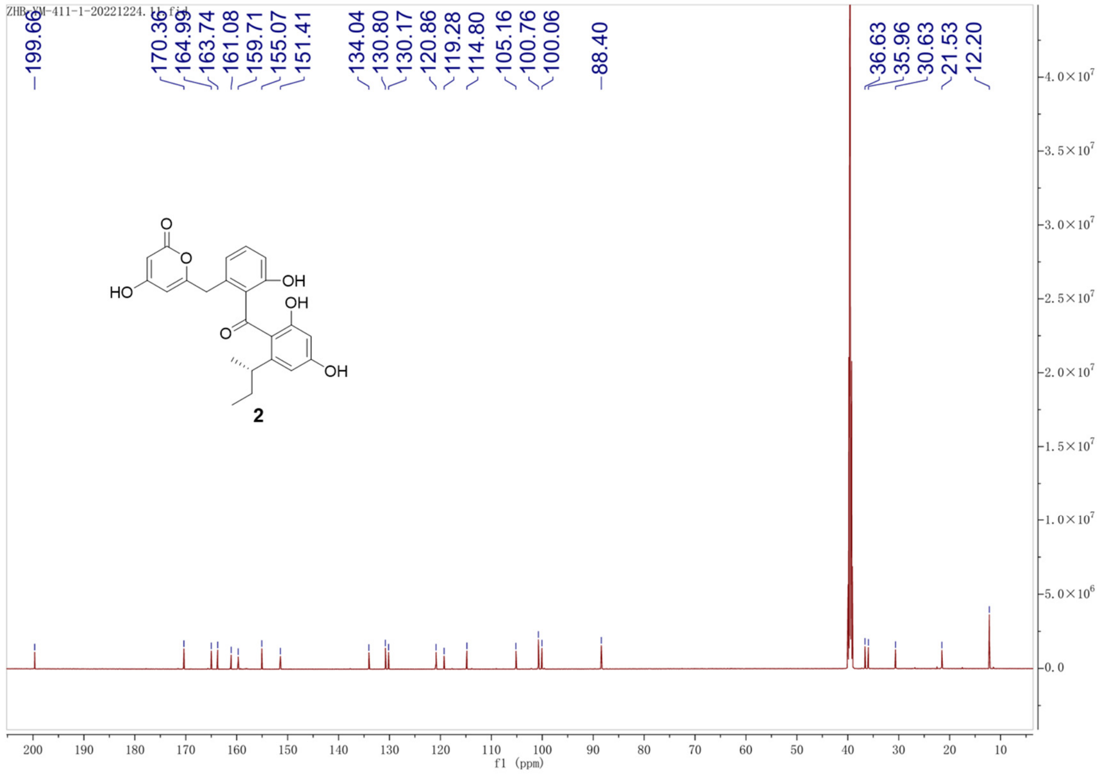

Figure S13. ^{13}C NMR spectrum (150 MHz) of verrucone B (**2**) in DMSO-d_6.

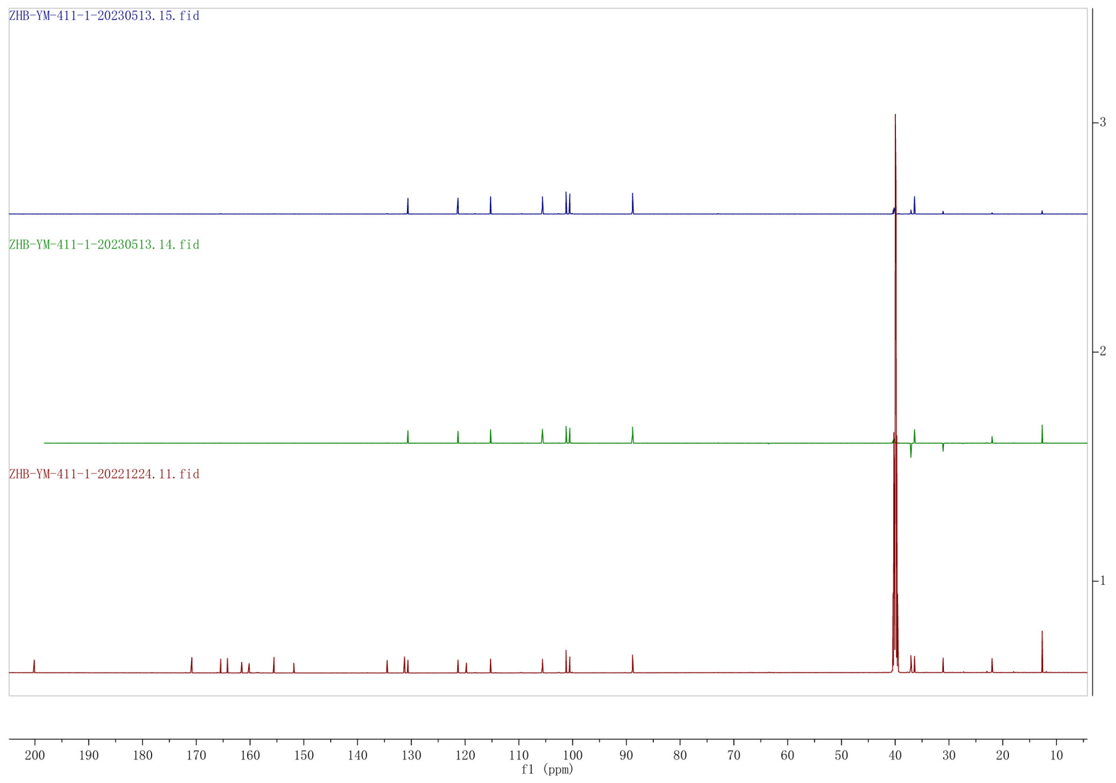

Figure S14. DEPT spectrum (150 MHz) of verrucone B (**2**) in DMSO-d_6.

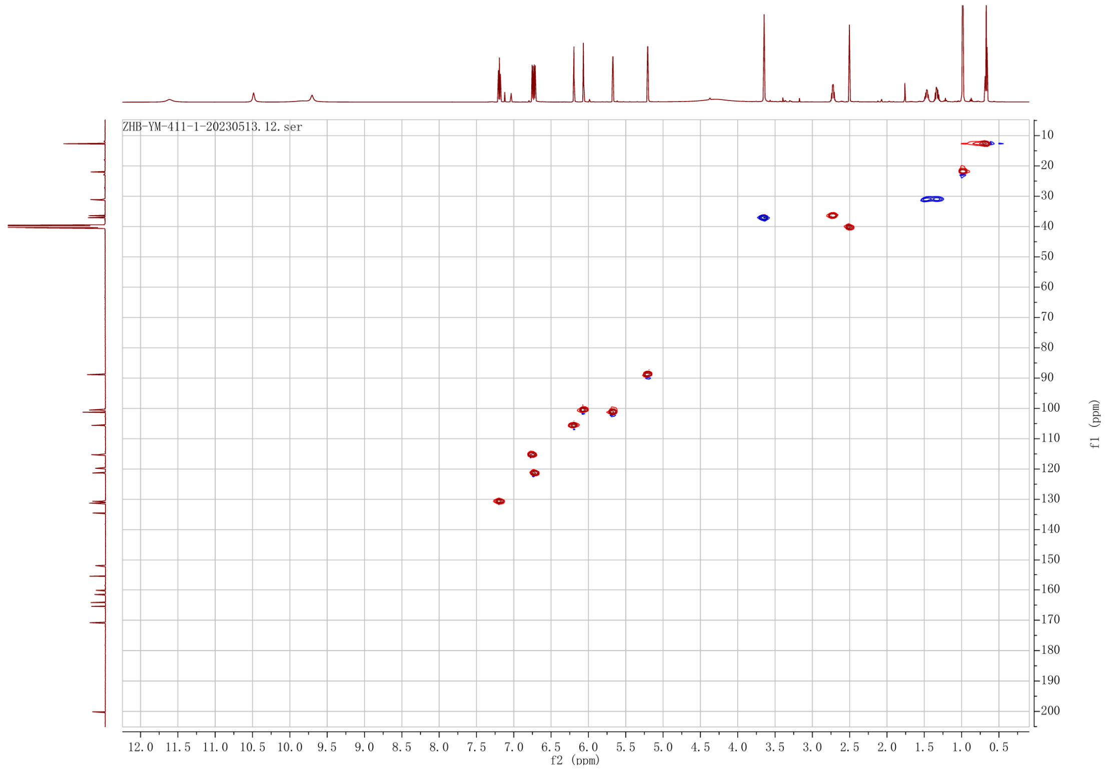

Figure S15. HSQC spectrum (150 MHz/600 MHz) of verrucone B (**2**) in DMSO-d_6.

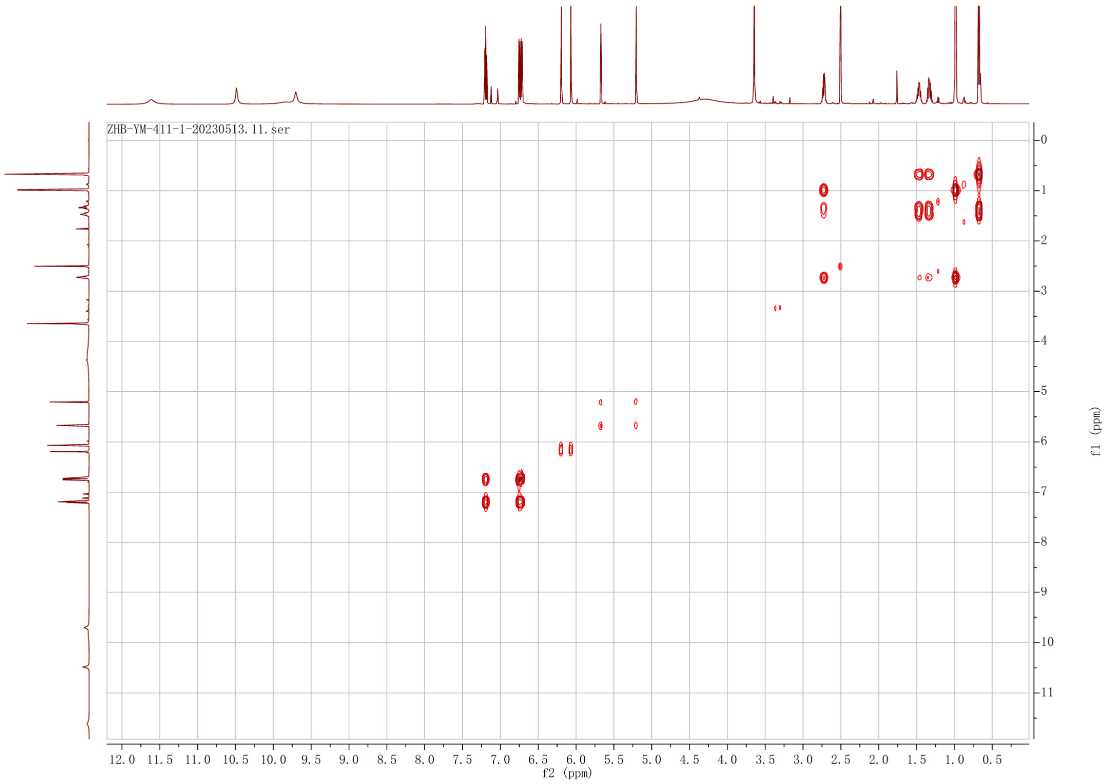

Figure S16. ^1H-^1H COSY spectrum (600 MHz) of verrucone B (**2**) in DMSO-d_6.

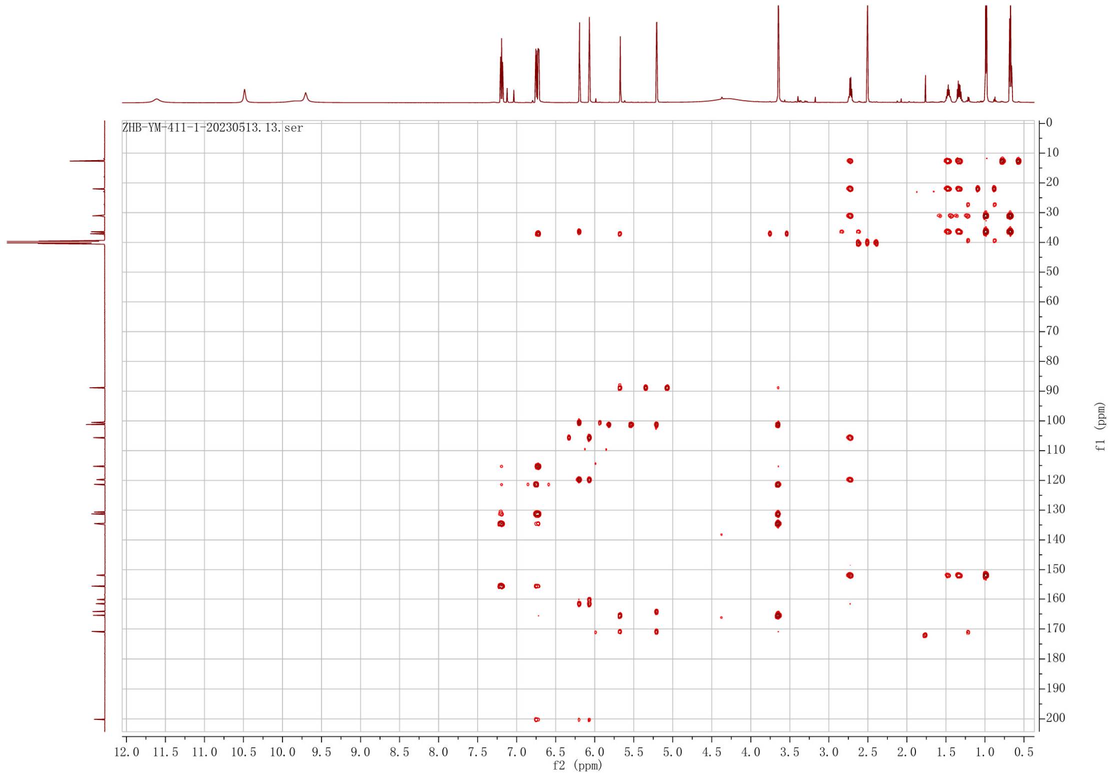

Figure S17. HMBC spectrum (150 MHz/600 MHz) of verrucone B (**2**) in DMSO-d_6.

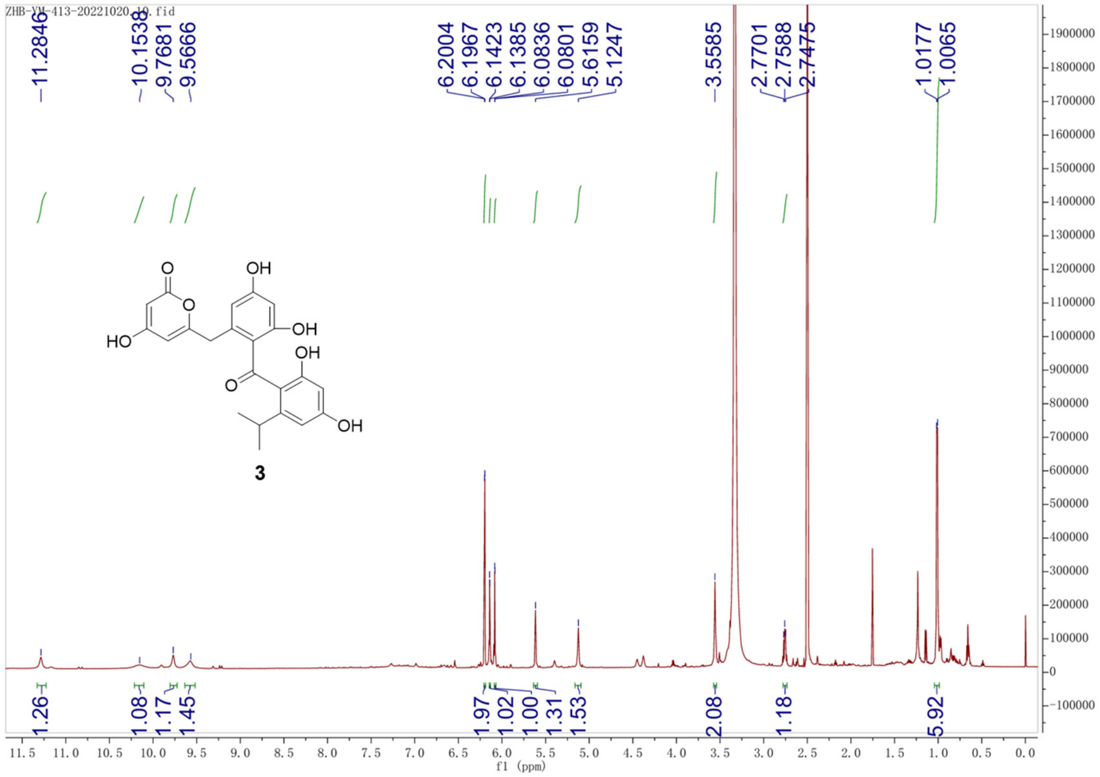

Figure S18. ^{1}H NMR spectrum (600 MHz) of verrucone C (**3**) in DMSO-d_6.

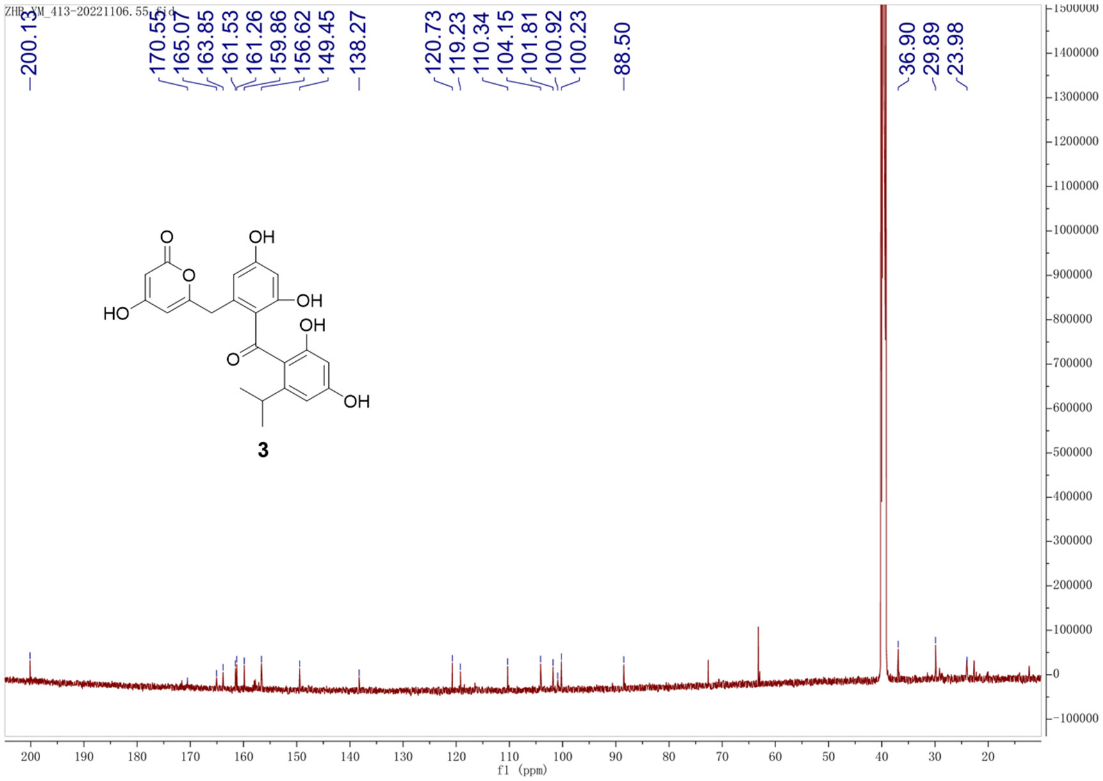

Figure S19. ^{13}C NMR spectrum (150 MHz) of verrucone C (**3**) in DMSO-d_6.

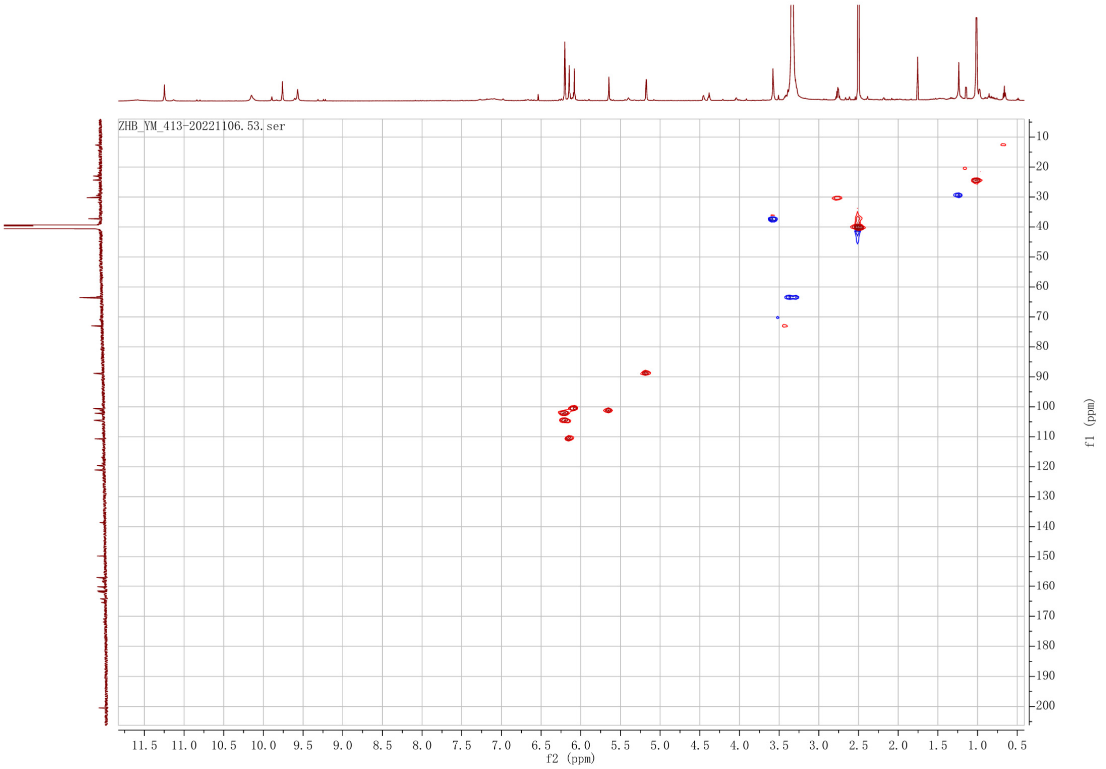

Figure S20. HSQC spectrum (150 MHz/600 MHz) of verrucone C (**3**) in DMSO-*d*6.

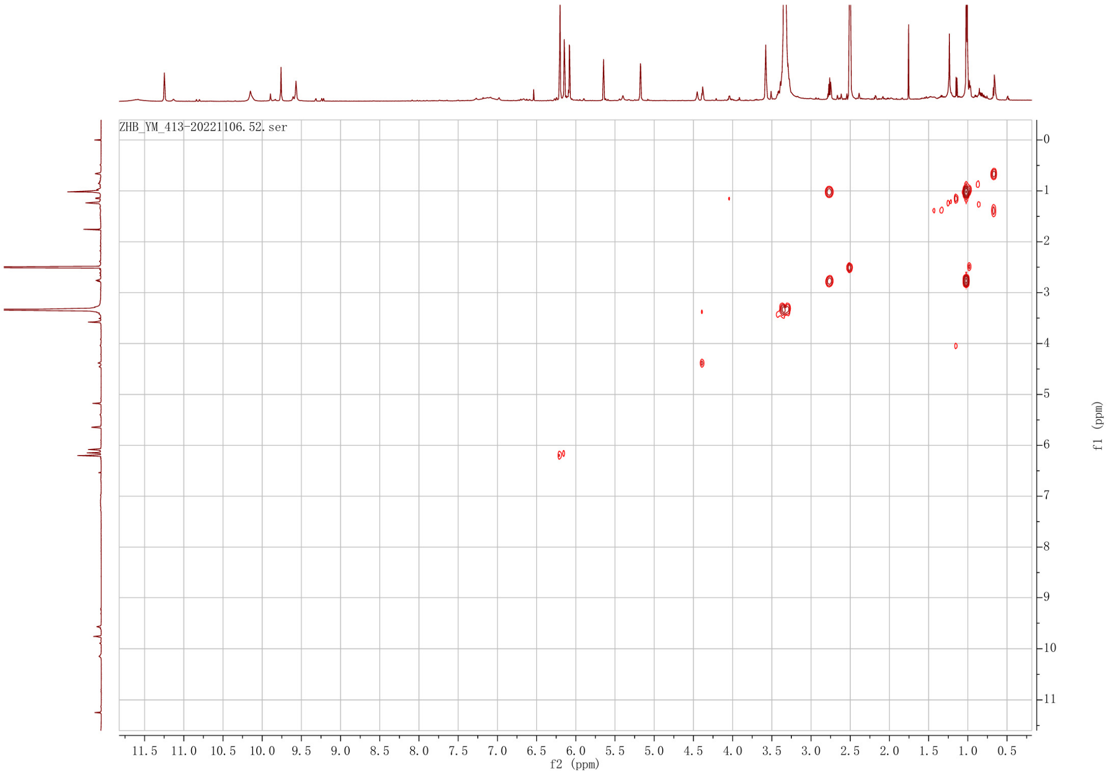

Figure S21. ^{1}H-^{1}H COSY spectrum (600 MHz) of verrucone C (**3**) in DMSO-*d*6.

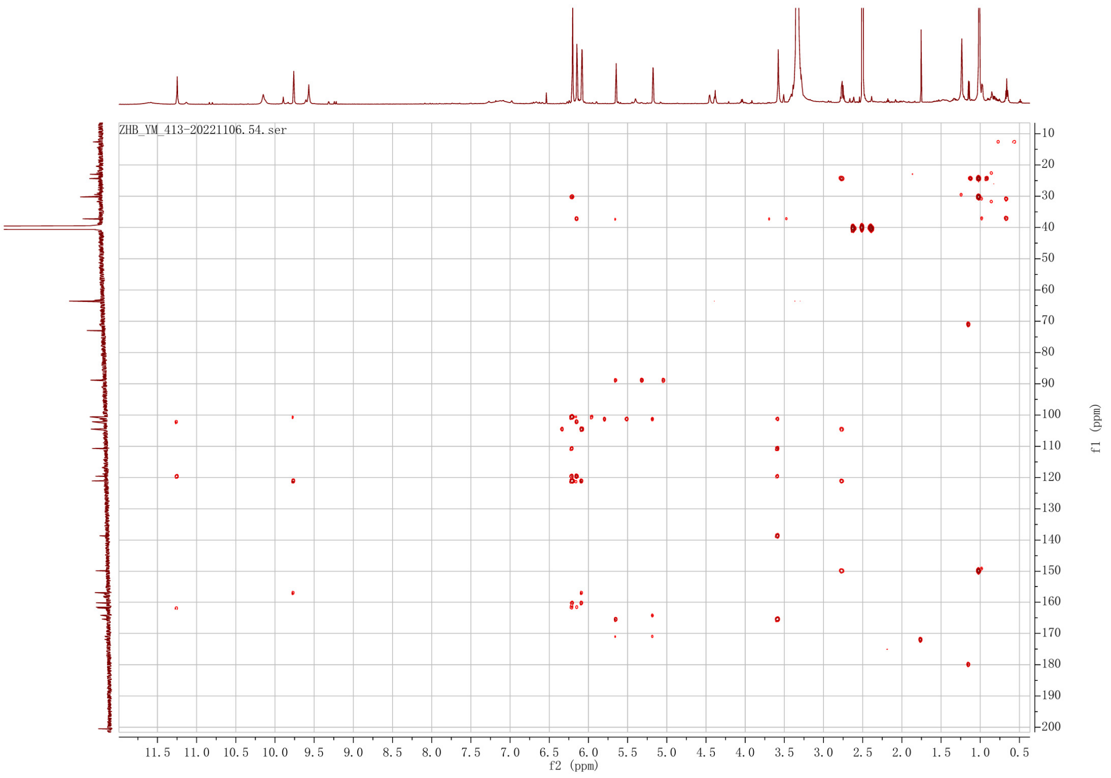

Figure S22. HMBC spectrum (150 MHz/600 MHz) of verrucone C (**3**) in DMSO-d_6.

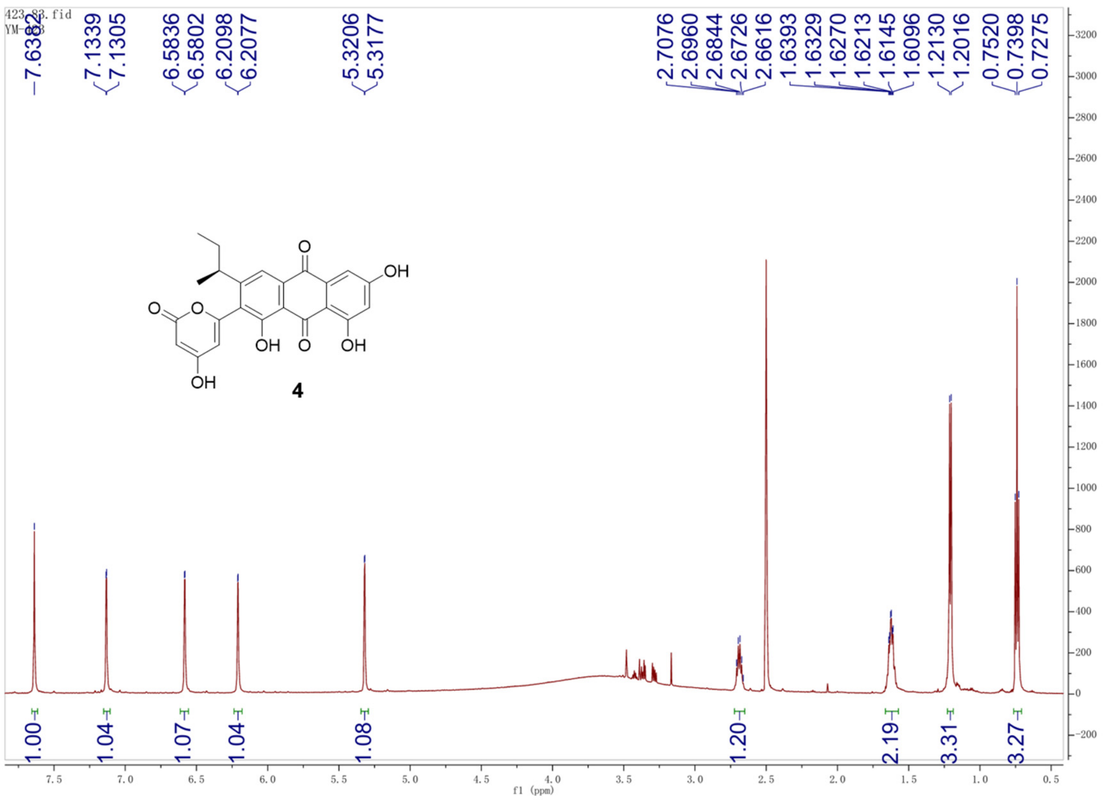

Figure S23. ^{1}H NMR spectrum (600 MHz) of verrucone D (**4**) in DMSO-d_6.

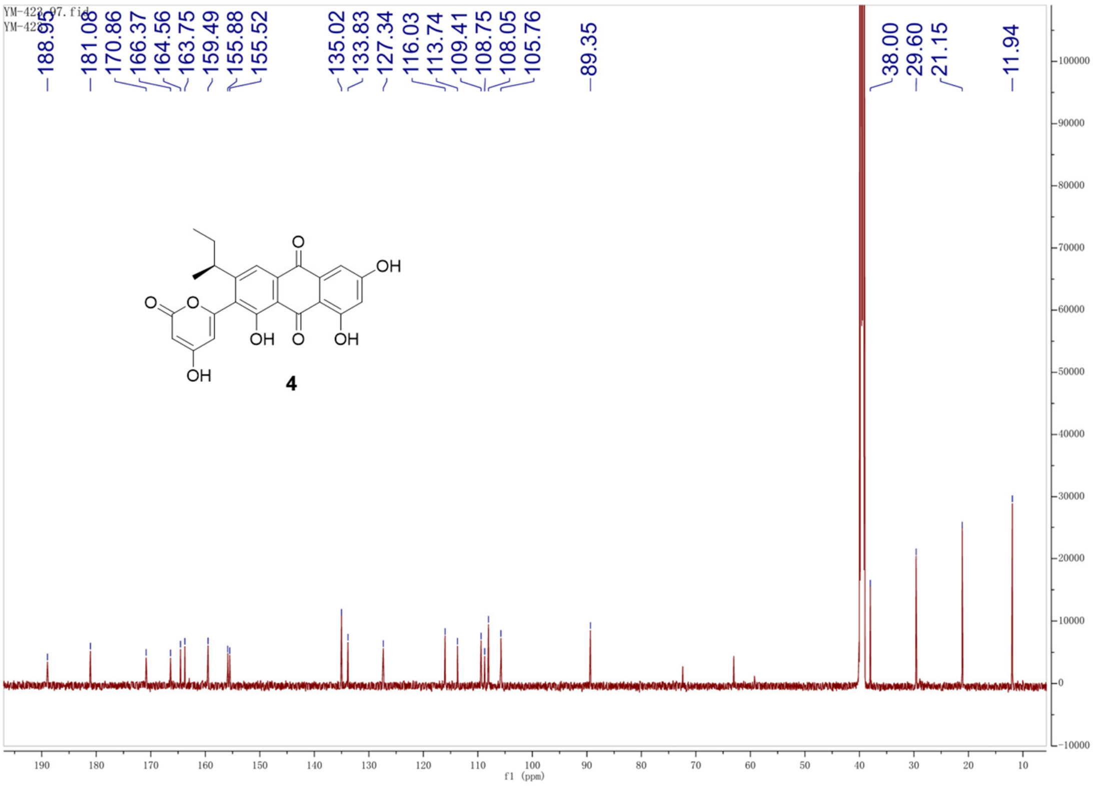

Figure S24. ^{13}C NMR spectrum (150 MHz) of verrucone D (**4**) in DMSO-*d*6.

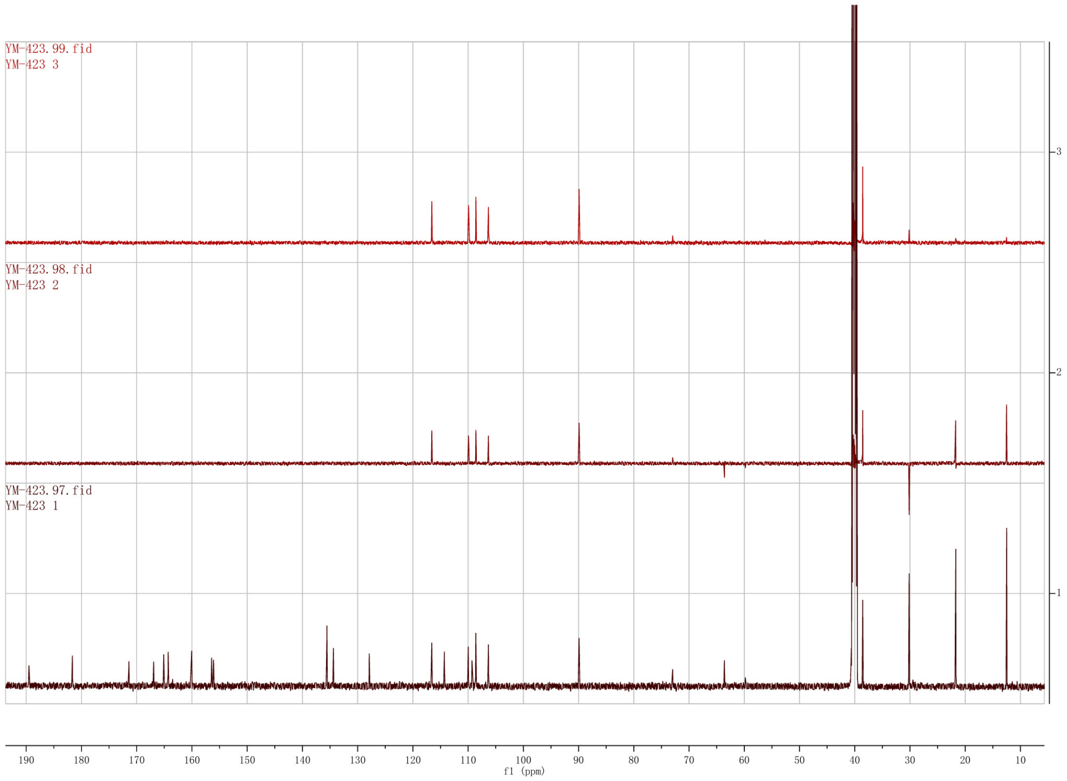

Figure S25. DEPT spectrum (150 MHz) of verrucone D (**4**) in DMSO-*d*6.

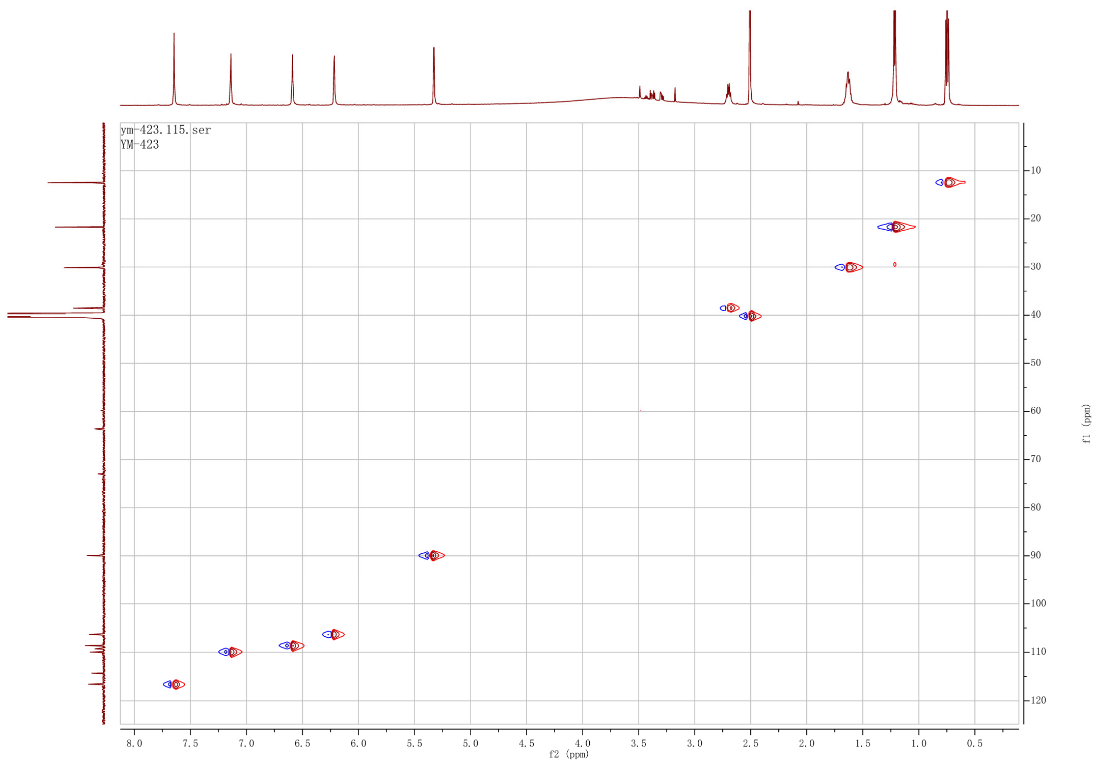

Figure S26. HSQC spectrum (150MHz/600 MHz) of verrucone D (**4**) in DMSO-d_6.

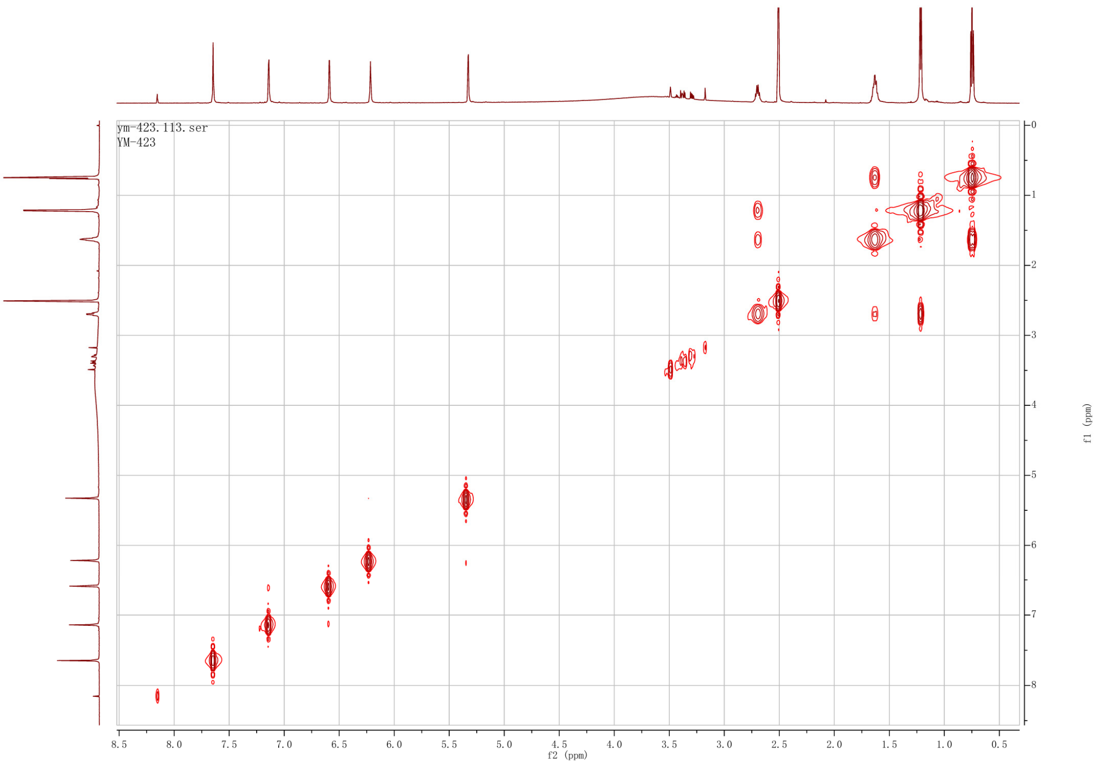

Figure S27. ^{1}H-^{1}H COSY spectrum (600 MHz) of verrucone D (**4**) in DMSO-d_6.

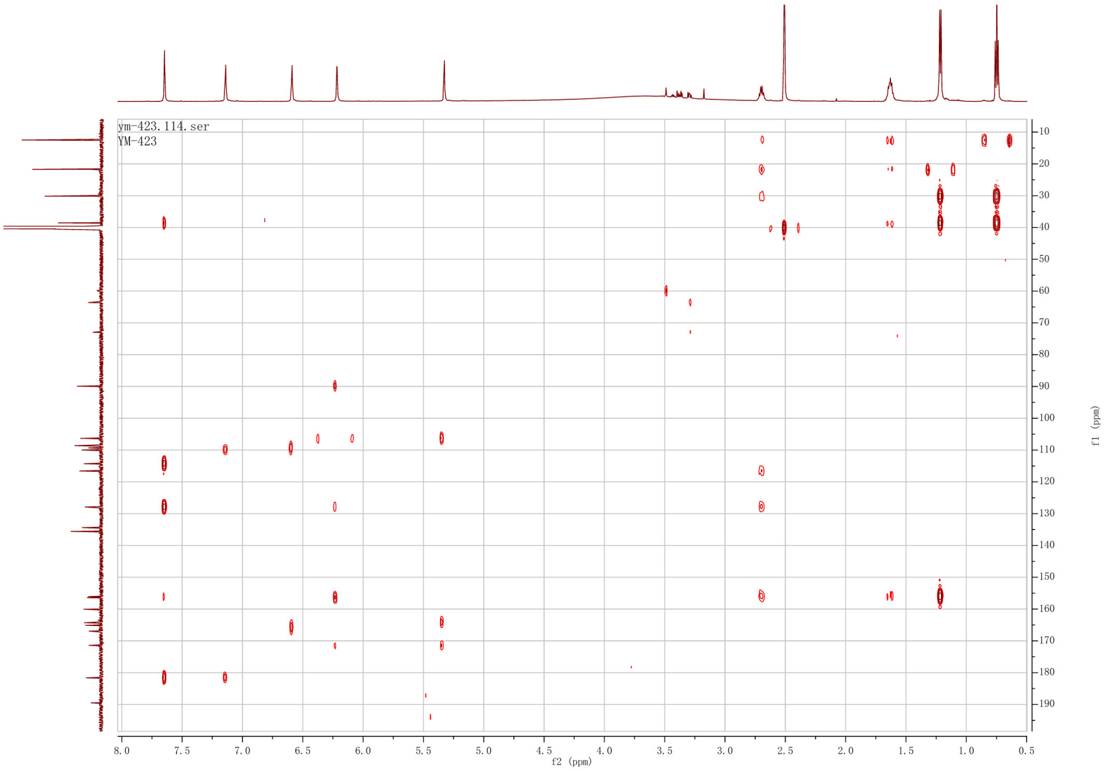

Figure S28. HMBC spectrum (150MHz/600 MHz) of verrucone D (**4**) in DMSO-d_6.

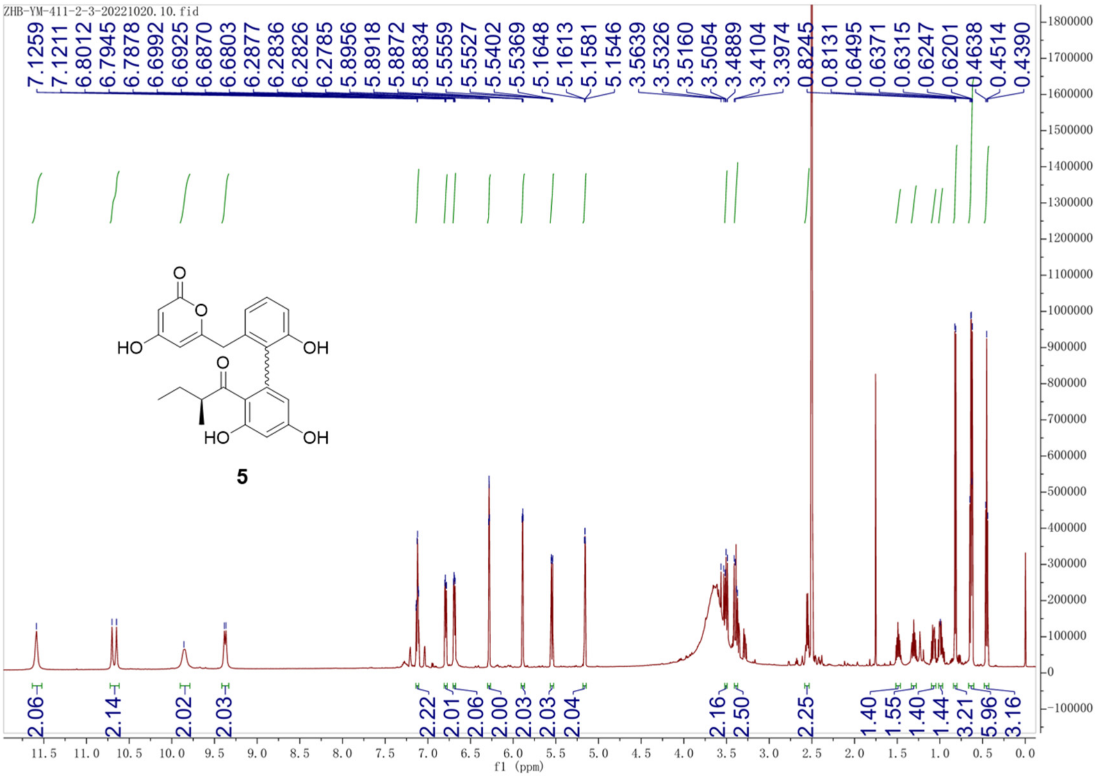

Figure S29. ^{1}H NMR spectrum (600 MHz) of verrucone E (**5**) in DMSO-d_6.

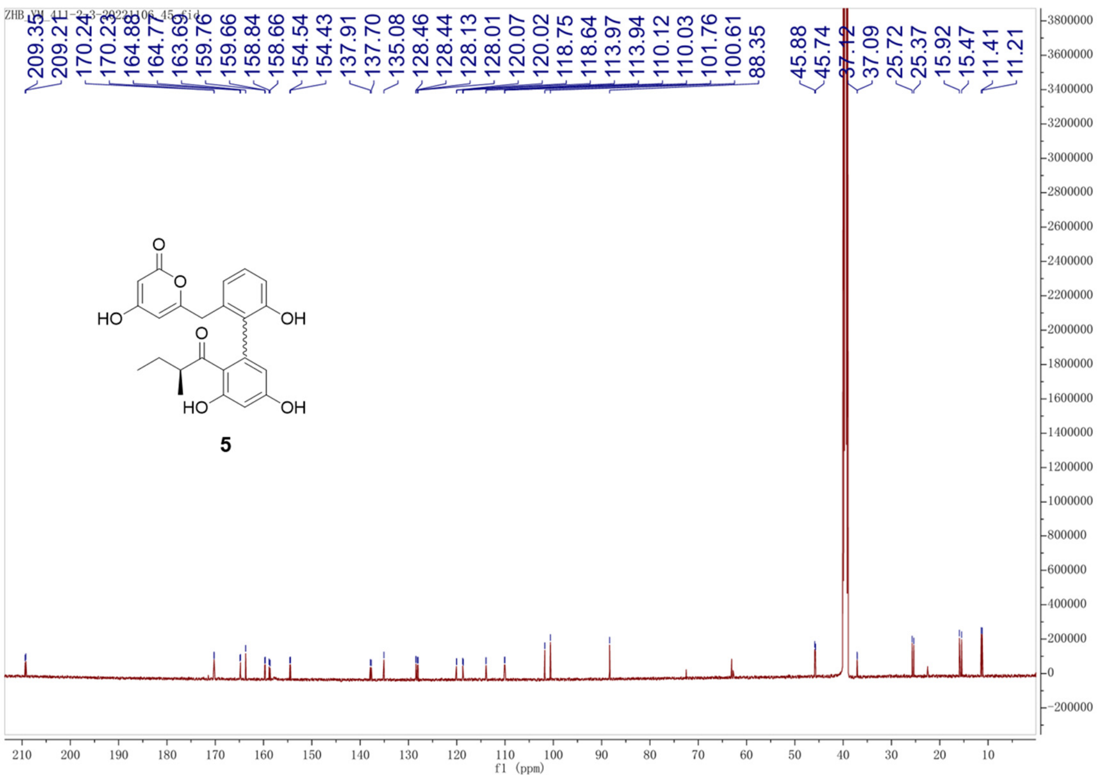

Figure S30. ^{13}C NMR spectrum (150 MHz) of verrucone E (**5**) in DMSO-d_6.

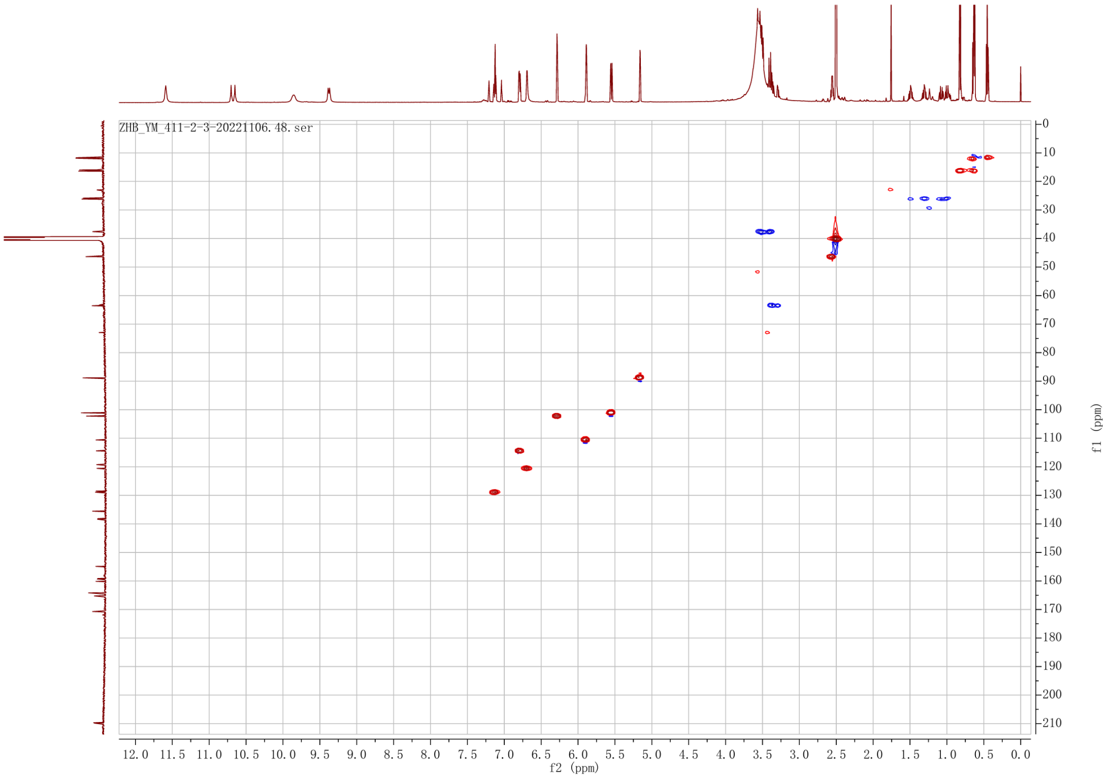

Figure S31. HSQC spectrum (150 MHz/600 MHz) of verrucone E (**5**) in DMSO-d_6.

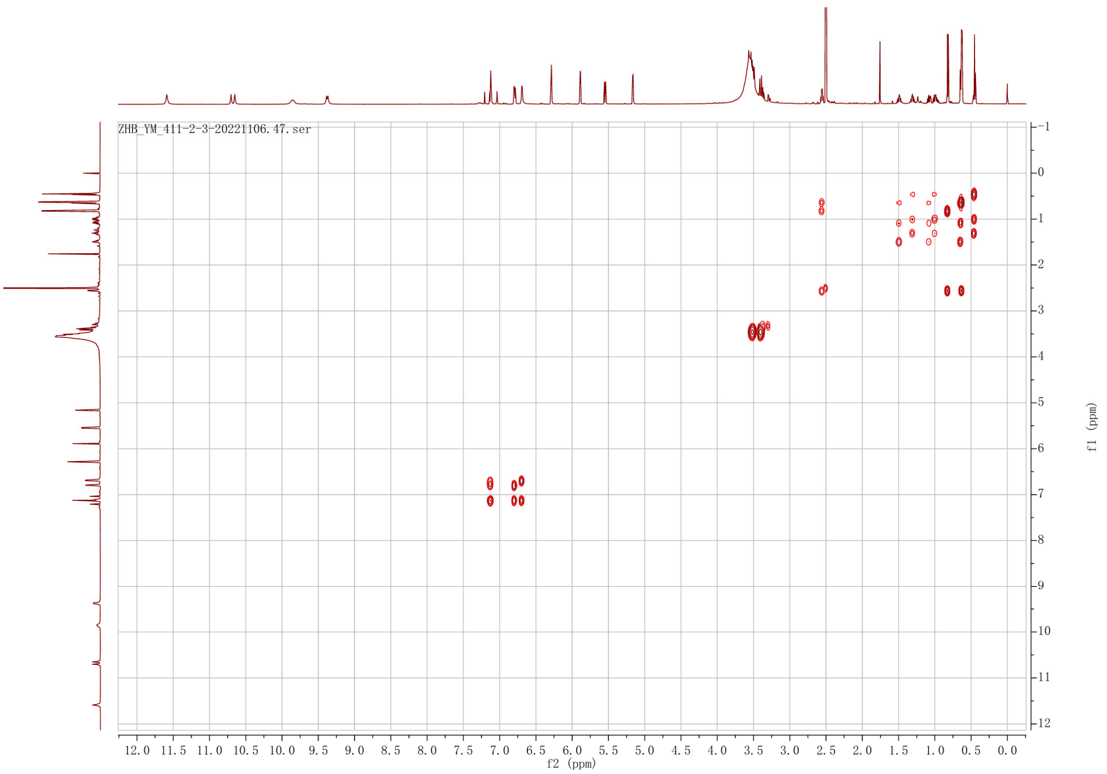

Figure S32. ^{1}H-^{1}H COSY spectrum (600 MHz) of verrucone E (**5**) in DMSO-d_6.

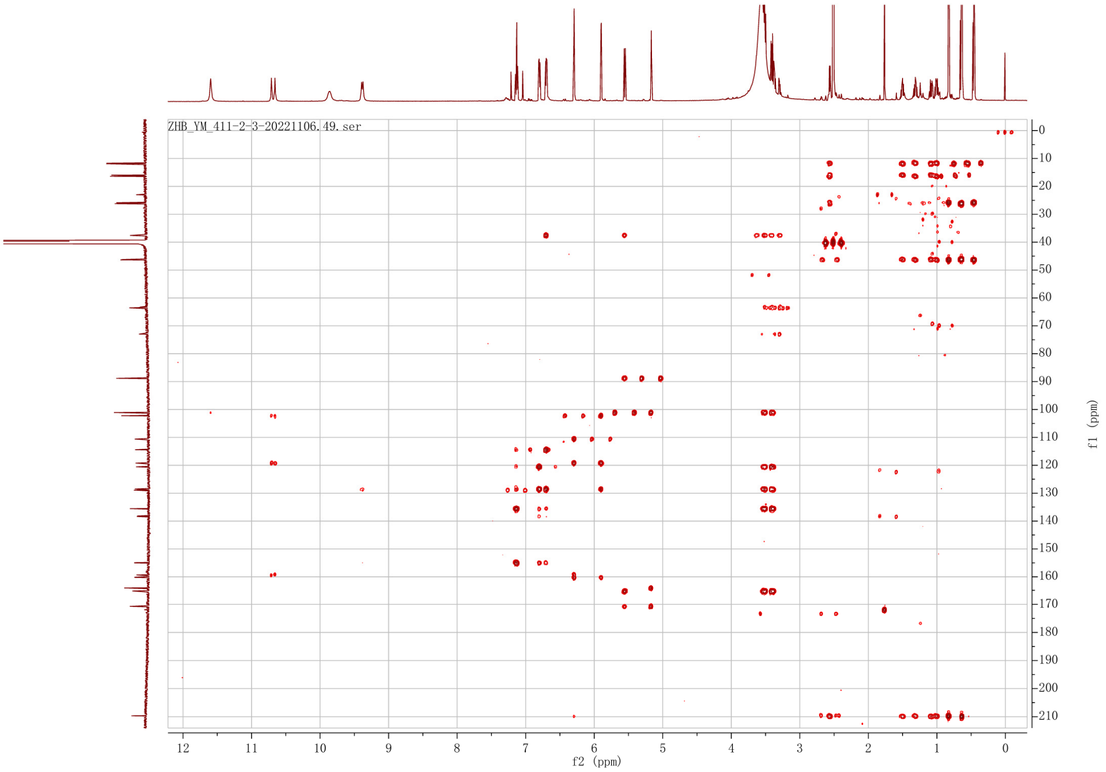

Figure S33. HMBC spectrum (150 MHz/600 MHz) of verrucone E (**5**) in DMSO-d_6.

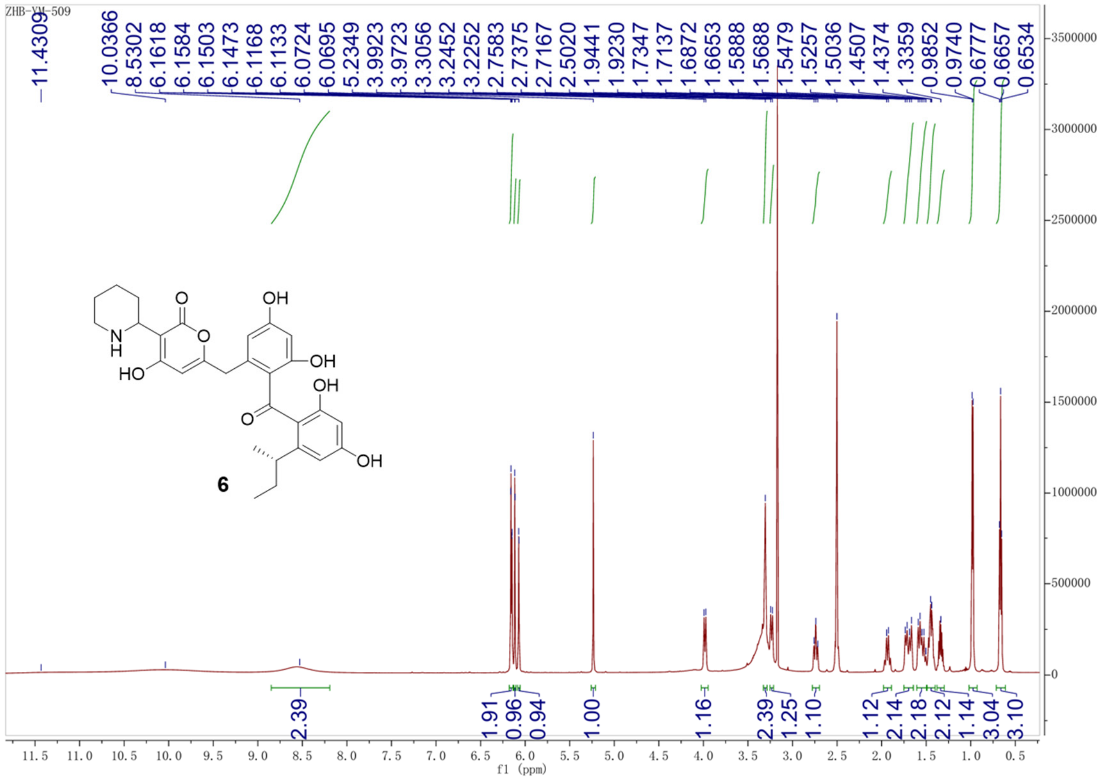

Figure S34. ^{1}H NMR spectrum (600 MHz) of verrucone F (**6**) in DMSO-d_6.

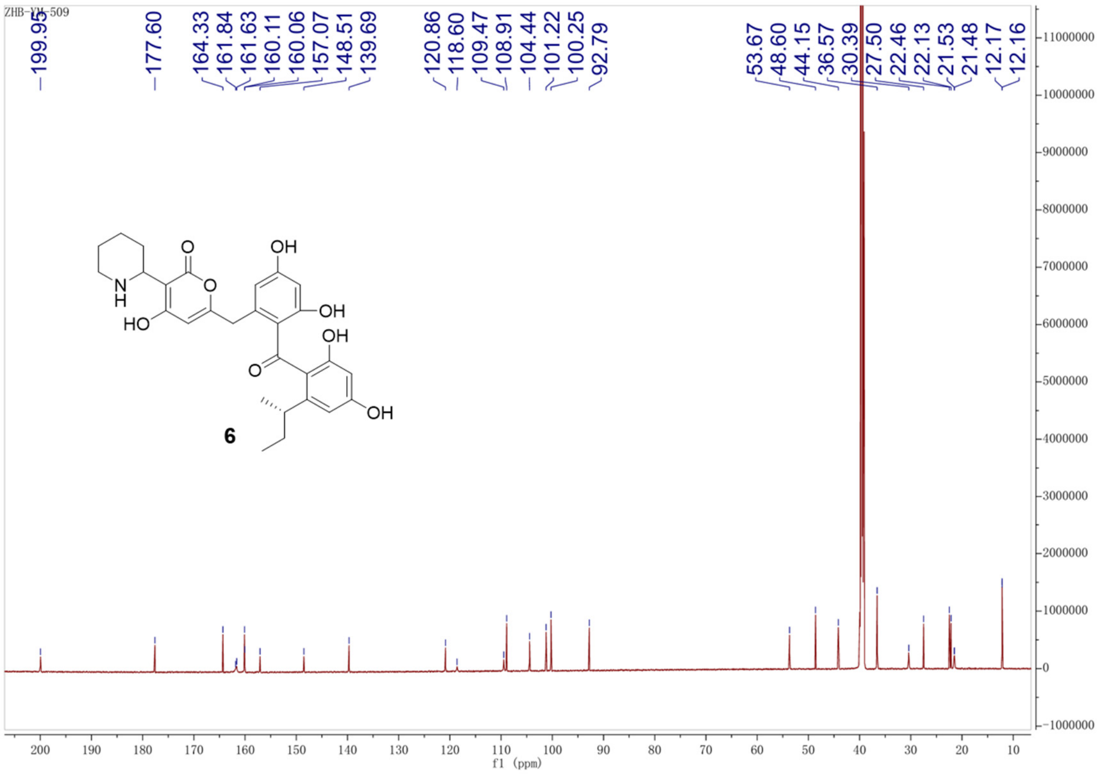

Figure S35. ^{13}C NMR spectrum (150 MHz) of verrucone F (**6**) in DMSO-d_6.

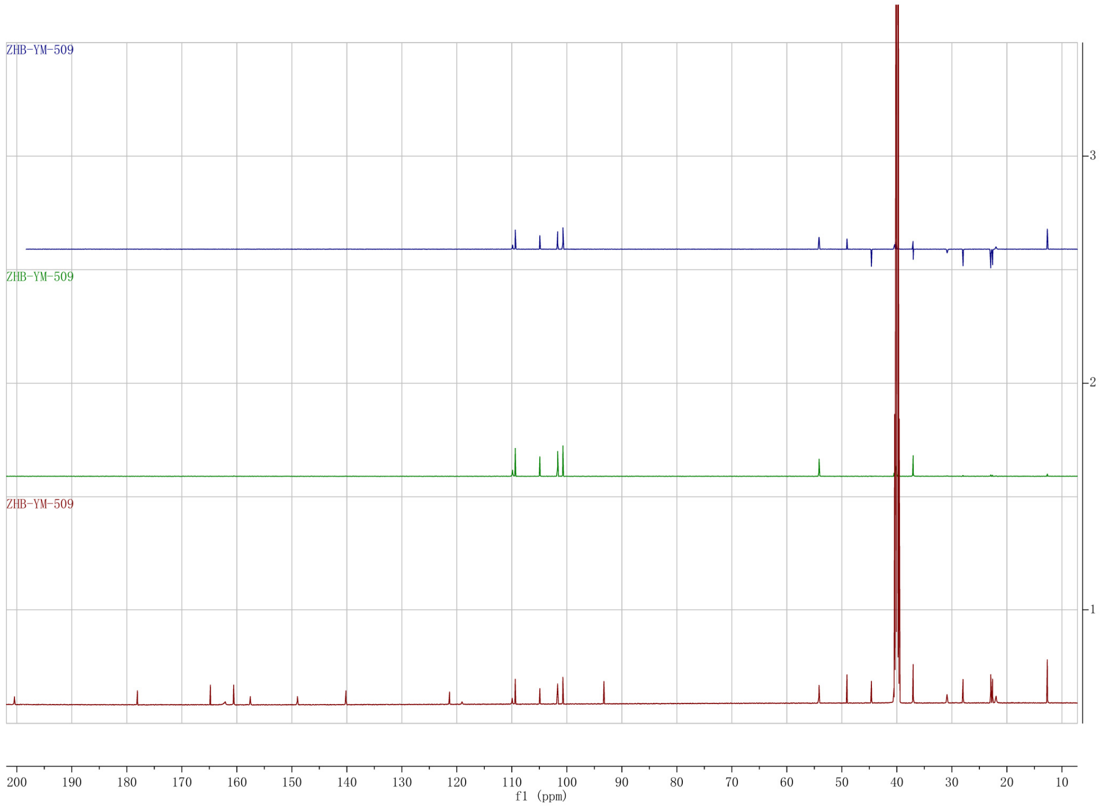

Figure S36. DEPT spectrum (150 MHz) of verrucone F (**6**) in DMSO-d_6.

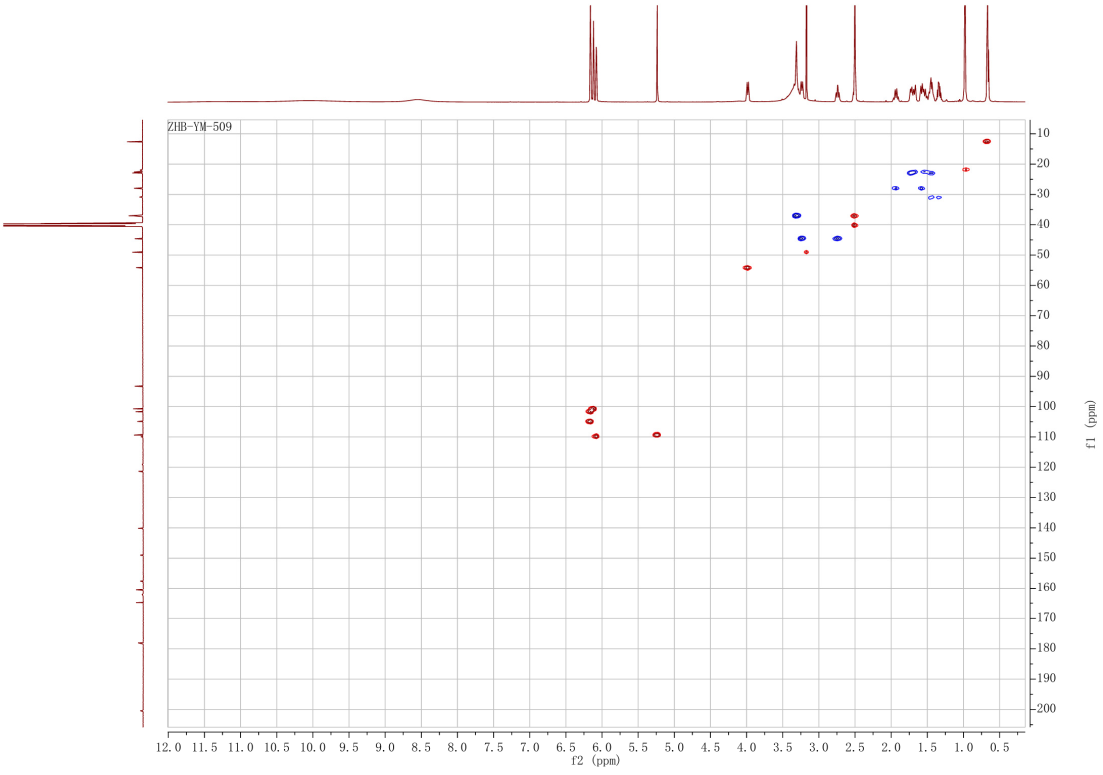

Figure S37. HSQC spectrum (150 MHz/600 MHz) of verrucone F (**6**) in DMSO-d_6.

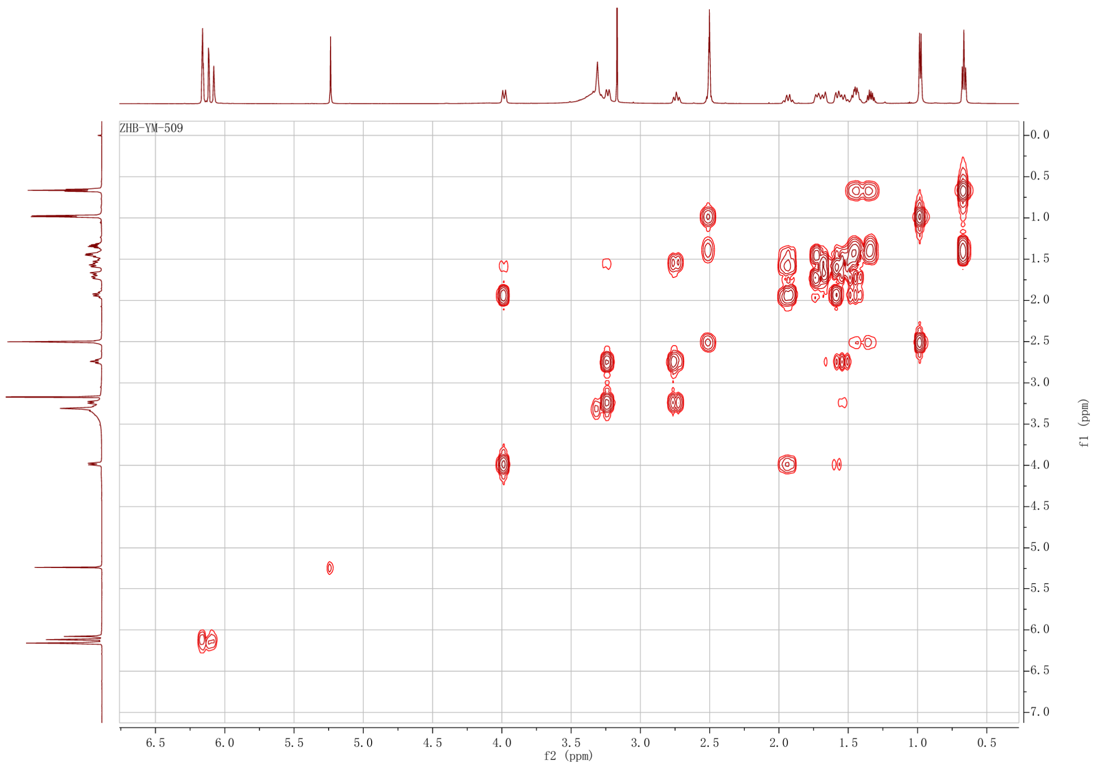

Figure S38. ^{1}H-^{1}H COSY spectrum (600 MHz) of verrucone F (**6**) in DMSO-d_6.

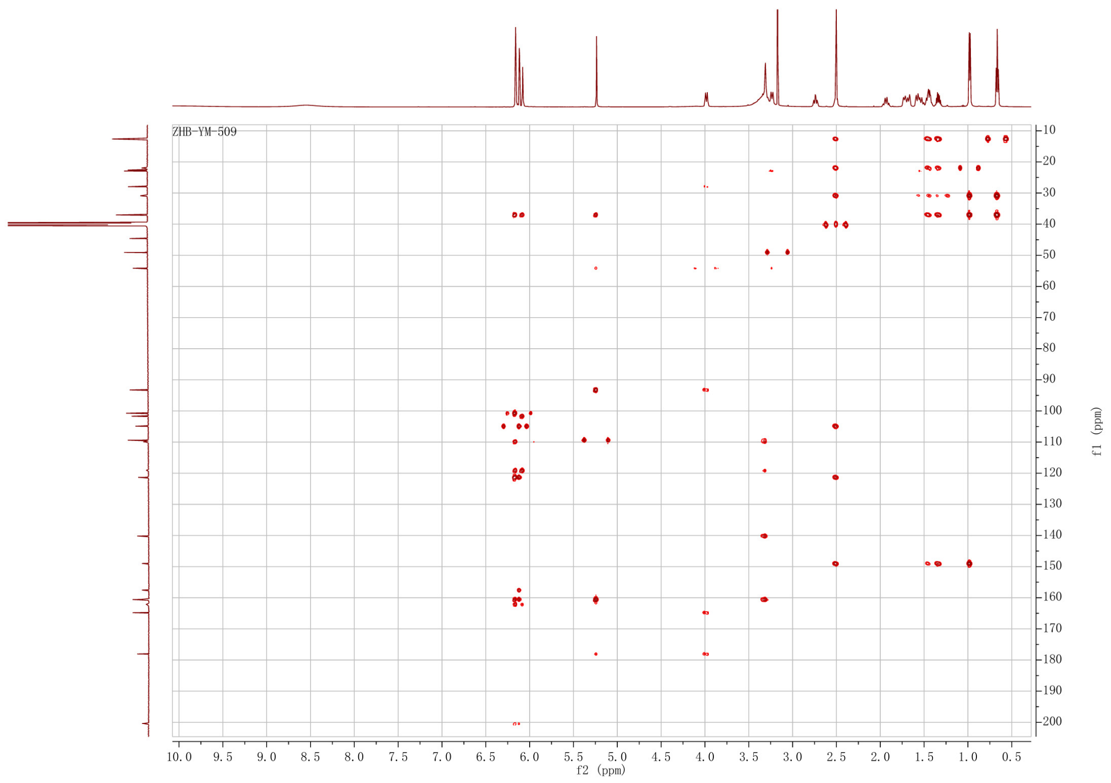

Figure S39. HMBC spectrum (150 MHz/600 MHz) of verrucone F (**6**) in DMSO-d_6.

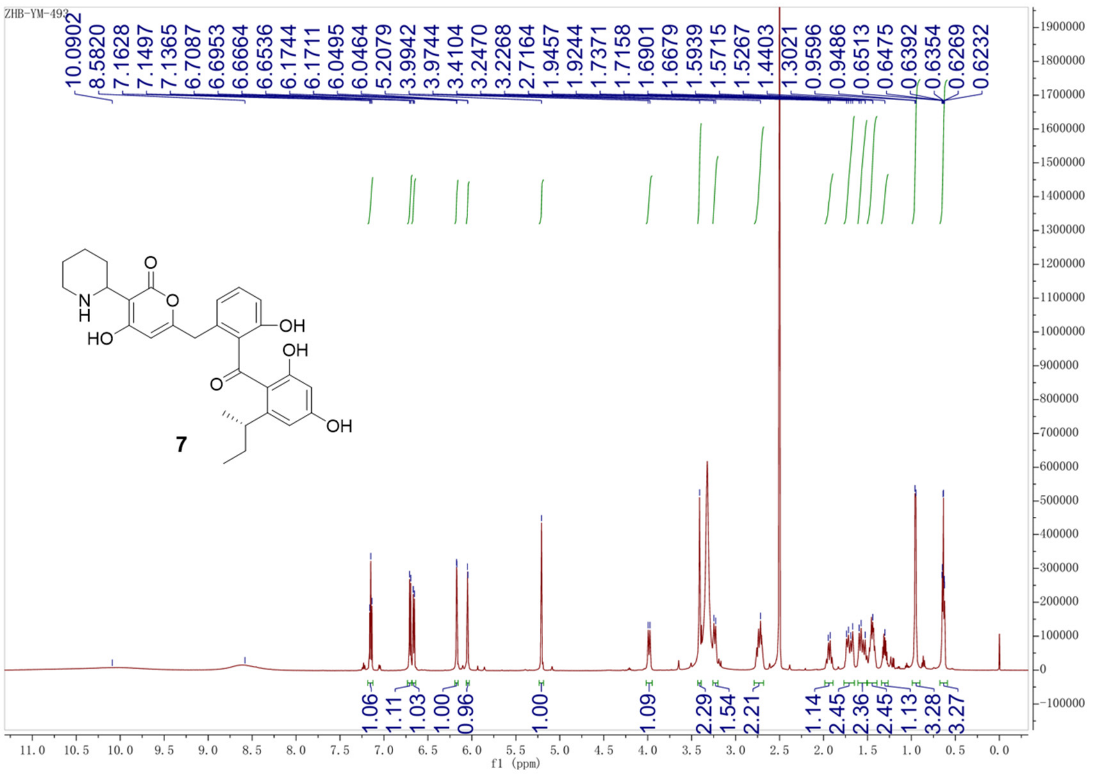

Figure S40. ^{1}H NMR spectrum (600 MHz) of verrucone G (**7**) in DMSO-d_6.

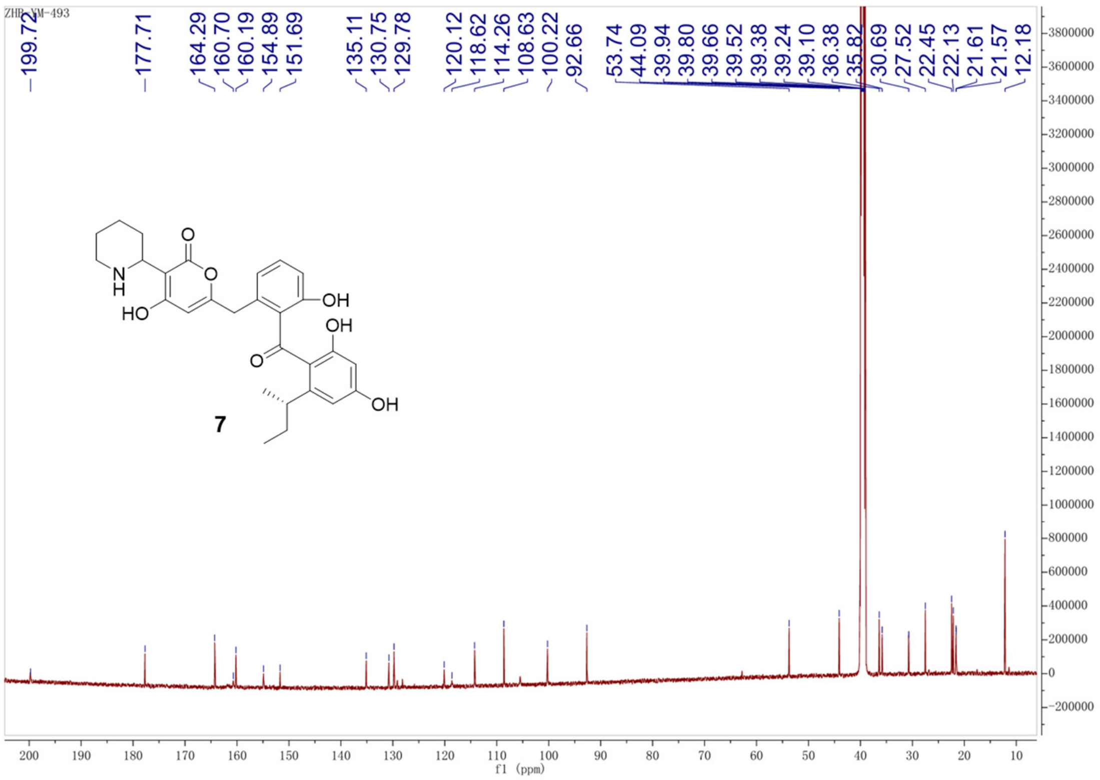

Figure S41. ^{13}C NMR spectrum (150 MHz) of verrucone G (**7**) in DMSO-d_6.

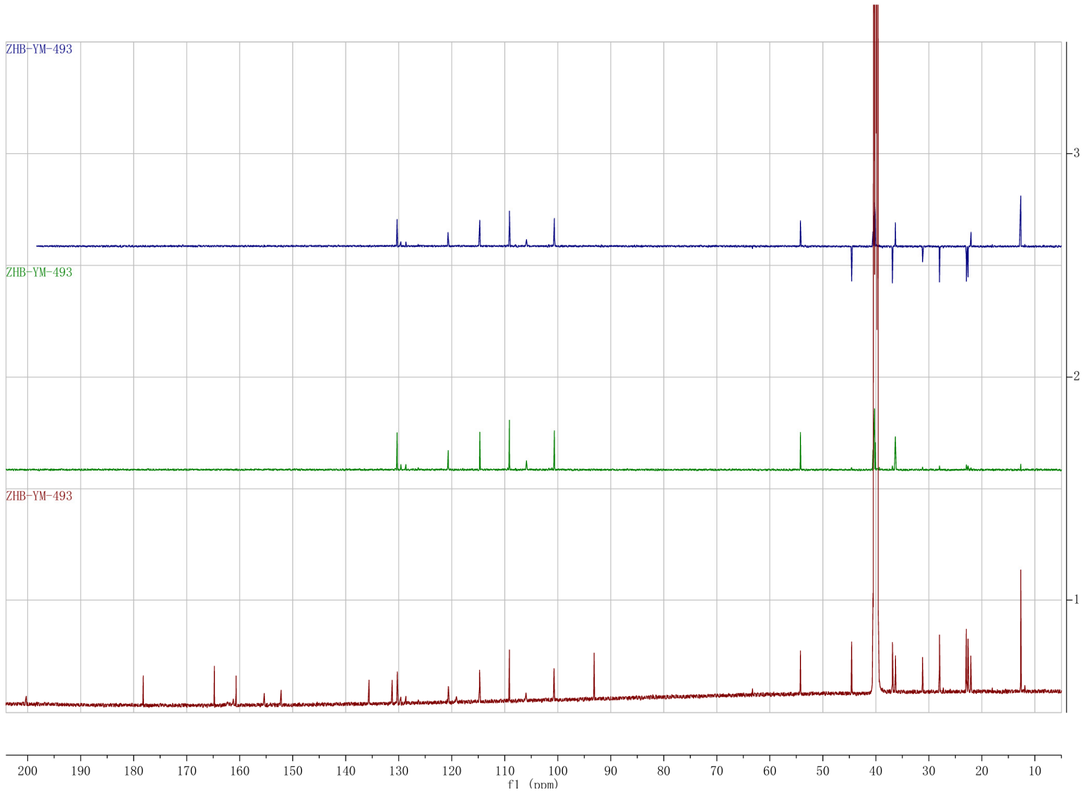

Figure S42. DEPT spectrum (150 MHz) of verrucone G (**7**) in DMSO-d_6.

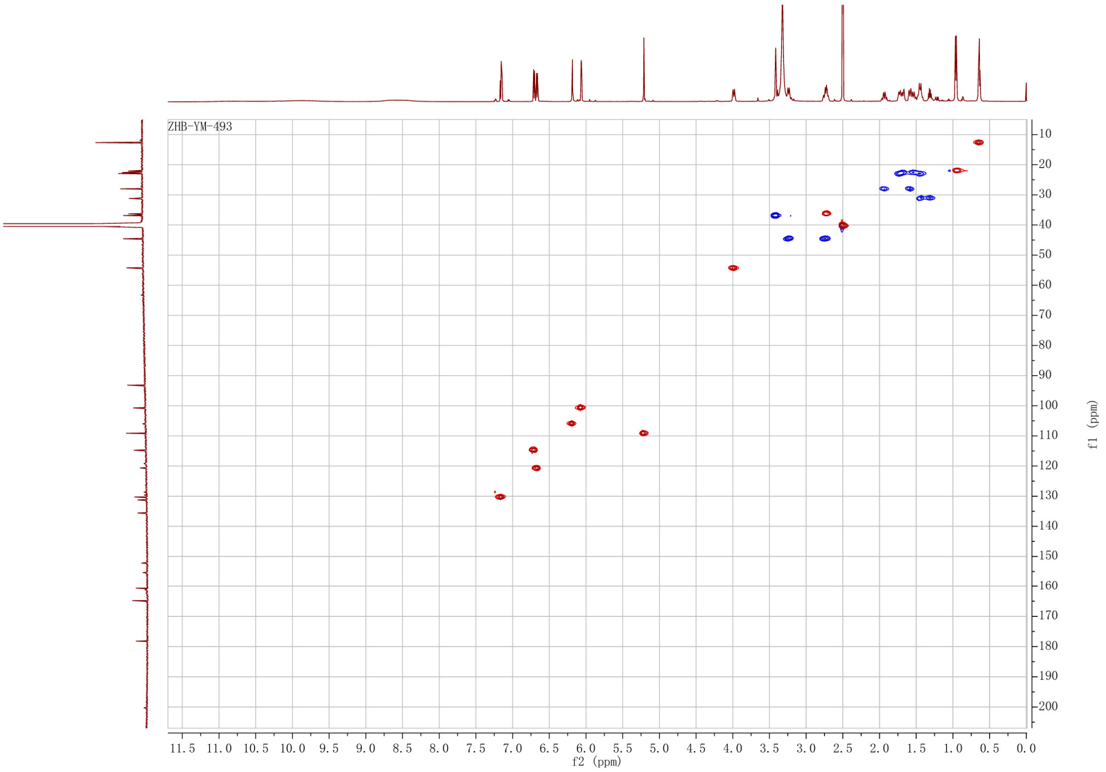

Figure S43. HSQC spectrum (150 MHz/600 MHz) of verrucone G (**7**) in DMSO-d_6.

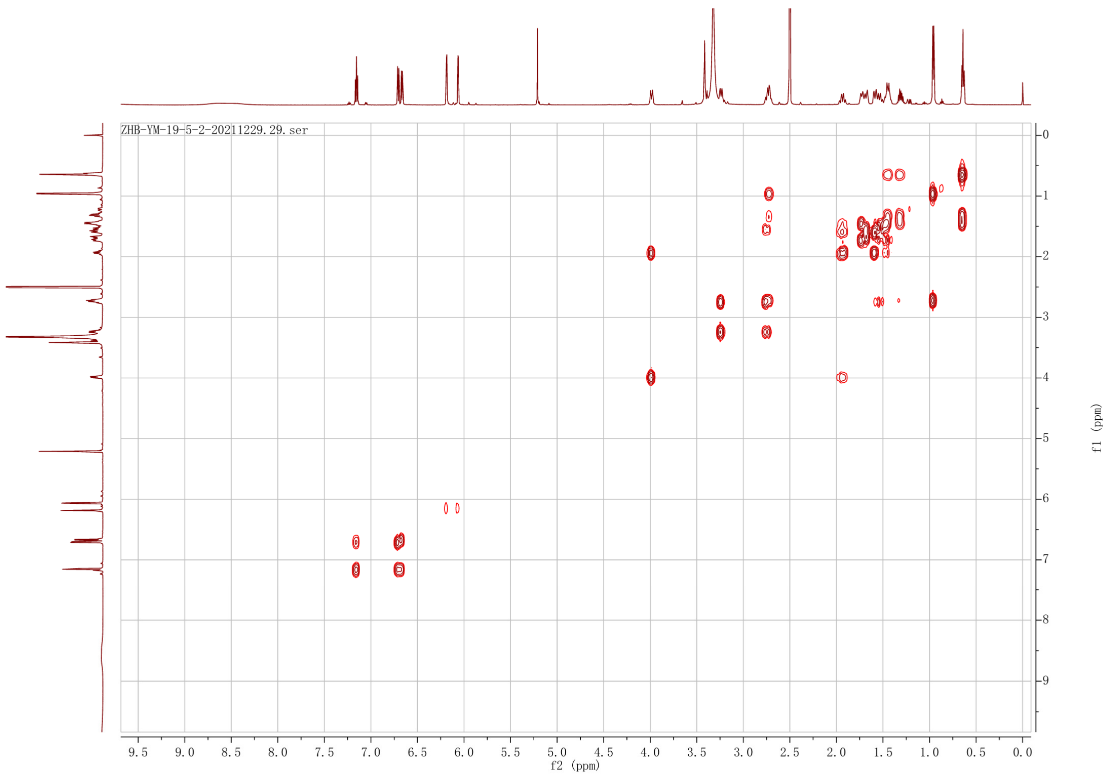

Figure S44. ^{1}H-^{1}H COSY spectrum (600 MHz) of verrucone G (**7**) in DMSO-*d*$_6$.

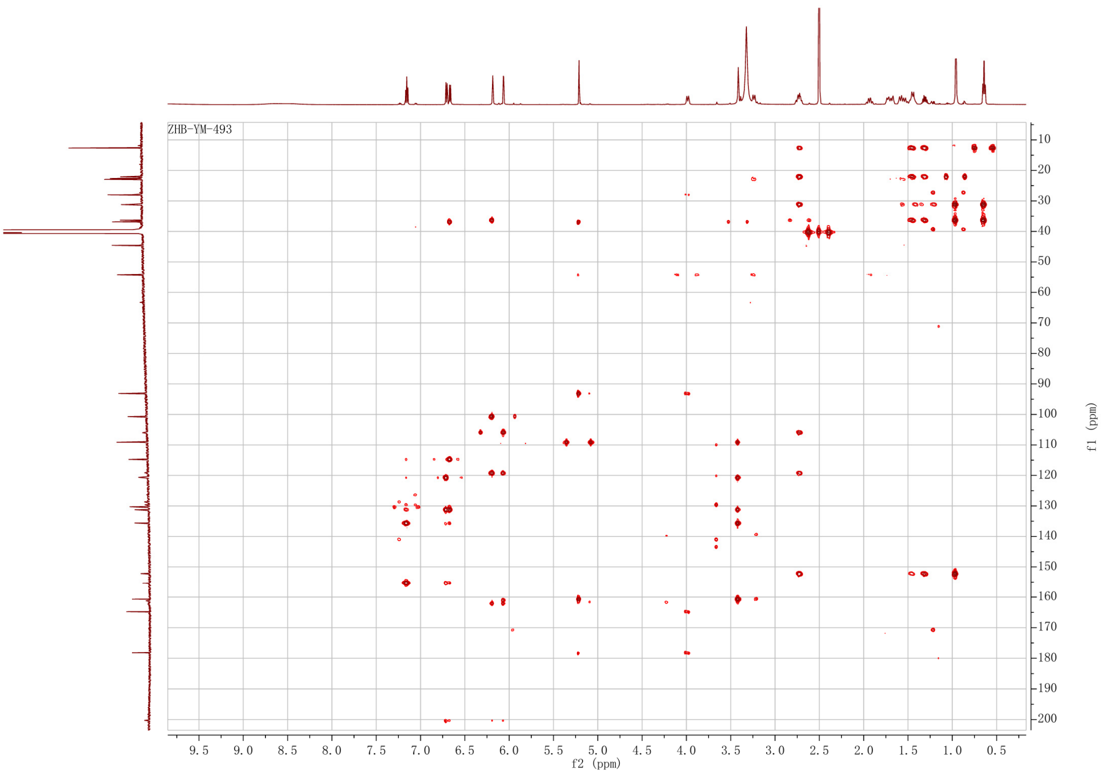

Figure S45. HMBC spectrum (150 MHz/600 MHz) of verrucone G (**7**) in DMSO-*d*$_6$.

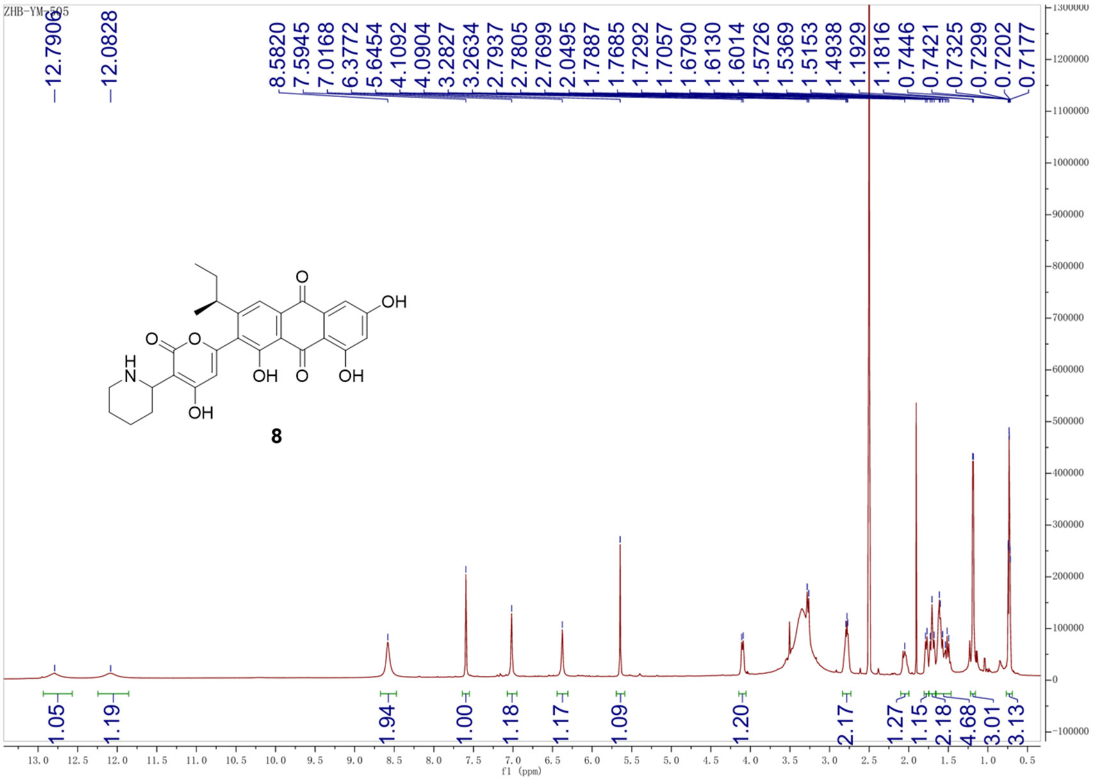

Figure S46. ^{1}H MNR spectrum (600 MHz) of verrucone H (**8**) in DMSO-d_6.

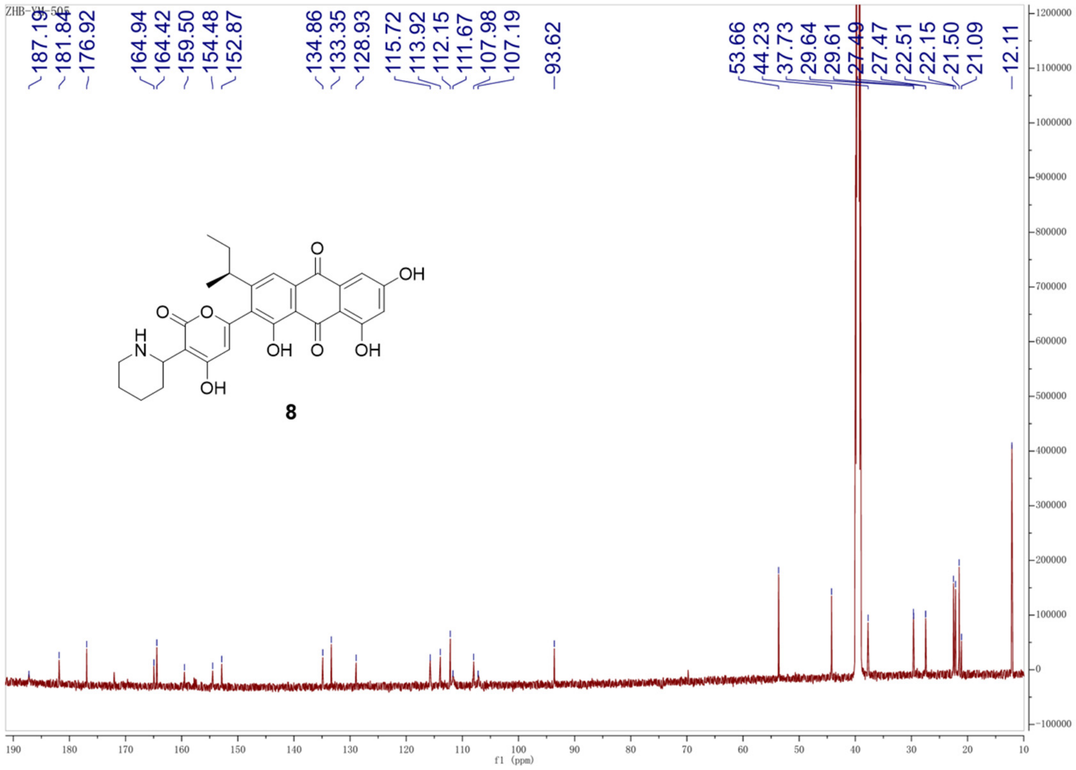

Figure S47. ^{13}C MNR spectrum (150 MHz) of verrucone H (**8**) in DMSO-d_6.

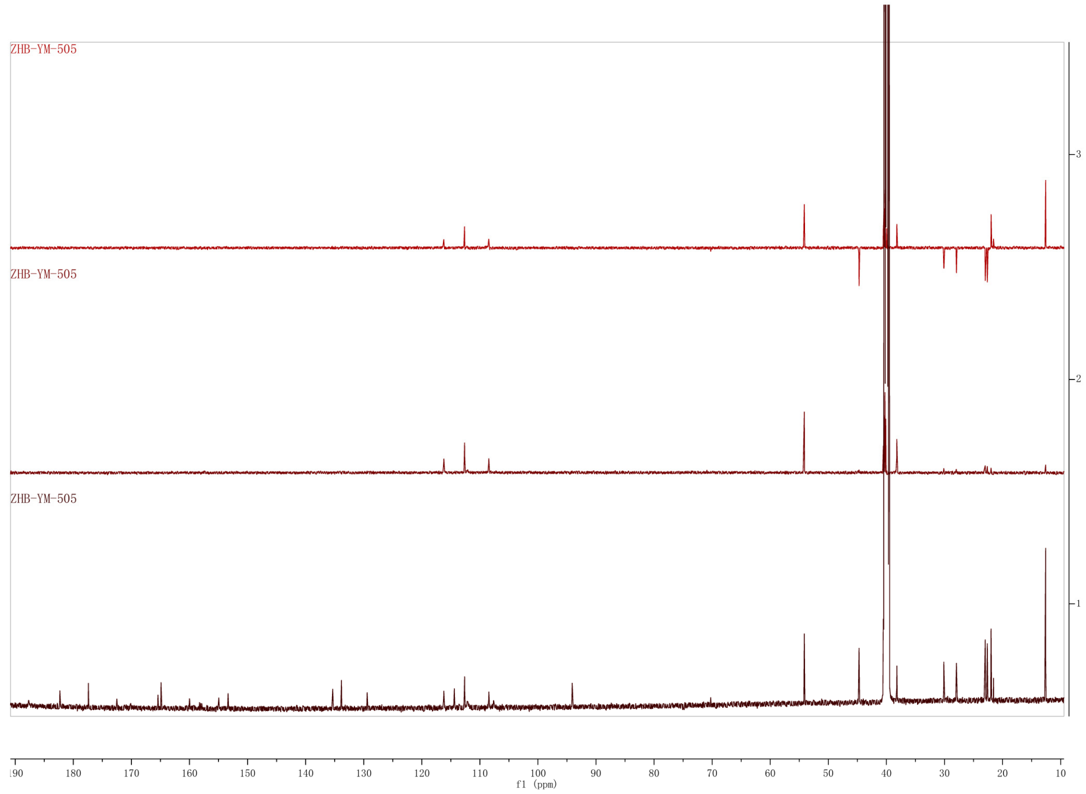

Figure S48. DEPT spectrum (150 MHz) of verrucone H (**8**) in DMSO-*d*6.

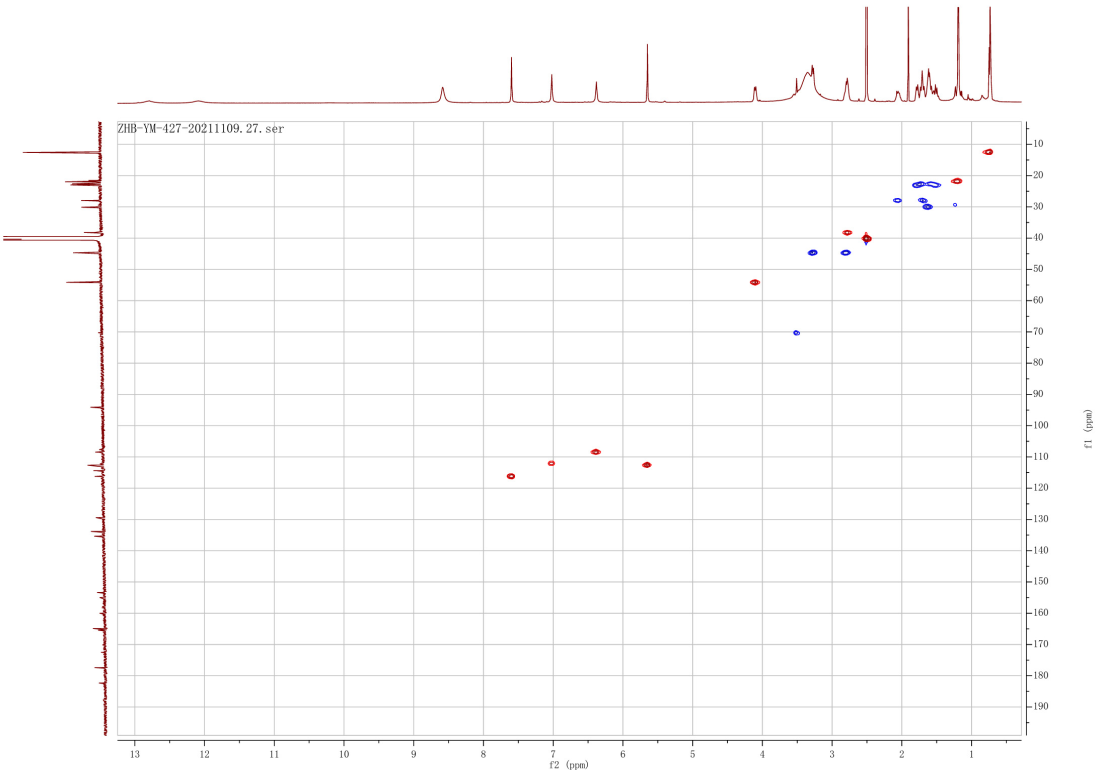

Figure S49. HSQC spectrum (150 MHz/600 MHz) of verrucone H (**8**) in DMSO-*d*6.

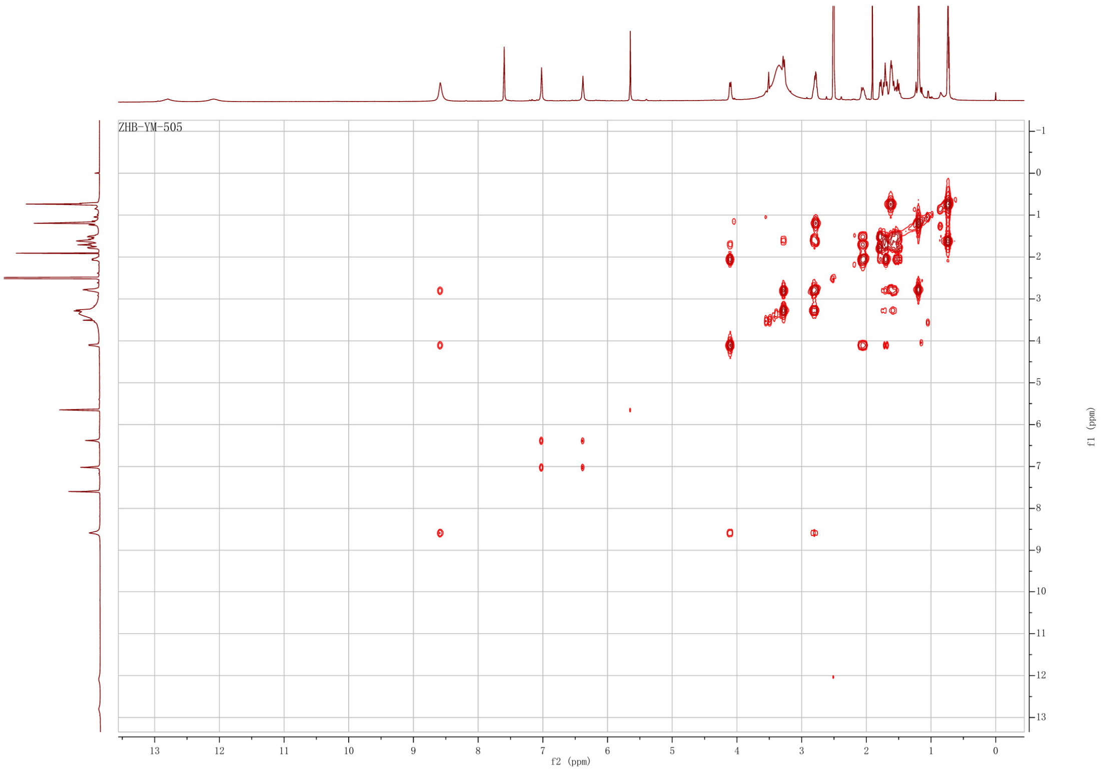

Figure S50. ^{1}H-^{1}H COSY spectrum (600 MHz) of verrucone H (**8**) in DMSO-*d*$_6$.

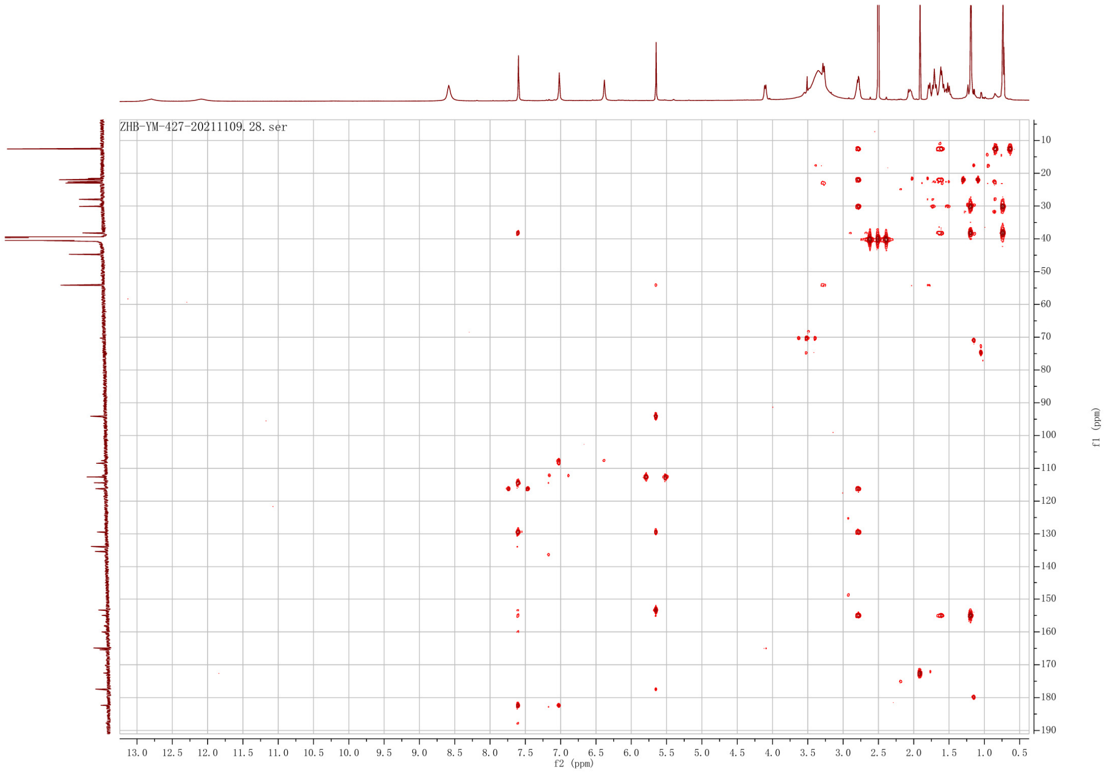

Figure S51. HMBC spectrum (150 MHz/600 MHz) of verrucone H (**8**) in DMSO-*d*$_6$.
